# Evaluating Synthetic Cyber Deception Strategies Under Uncertainty via Game Theory Approach: Linking Information Leakage and Game Outcomes in Cyber Deception

**DOI:** 10.3390/s26061748

**Published:** 2026-03-10

**Authors:** Mohammad Shahin, Mazdak Maghanaki, Fengshan Frank Chen

**Affiliations:** 1 Department of Industrial and Systems Engineering, University of Tennessee, Knoxville, TN 37996, USA; 2 Department of Mechanical, Aerospace, and Industrial Engineering, University of Texas, San Antonio, TX 78249, USAff.chen@utsa.edu (F.F.C.)

**Keywords:** game theory, cyber deception, Bayesian Stackelberg security games, strong Stackelberg equilibrium, decoy (honeypot) allocation optimization, value of deception and price of transparency metrics, quantal response, information leakage, deception capacity, network cybersecurity

## Abstract

The study develops a game-theoretic evaluation framework for cyber deception that quantifies deception benefit relative to an otherwise matched non-deceptive baseline and links strategic outcomes to information disclosure. A defender–attacker interaction is modeled through a paired design consisting of a baseline game without deception and a corresponding decoy-enabled deception game, enabling direct measurement of deception impact through two operational metrics: the value of deception, defined as the baseline-referenced change in defender equilibrium utility attributable to deception, and the price of transparency, defined as the marginal loss induced by increased observability of the true system state. The analysis characterizes defender-optimal deception strategies, derives interpretable bounds and break-even conditions under which deception becomes ineffective due to cost or detectability, and establishes approximation properties that support scalable allocation rules. To complement equilibrium-based evaluation, the study introduces an information-theoretic uncertainty construct that captures the extent to which deception preserves attacker uncertainty after observation, providing a mechanism-level interpretation of when and why value of deception degrades as transparency increases. Computational experiments across heterogeneous scenarios demonstrate consistent cross-setting comparability, reveal tradeoffs among decoy realism, budget, and attacker rationality, and identify regimes in which simplified allocation heuristics approach optimal performance.

## 1. Introduction

In the dynamic domain of cybersecurity, defenders are progressively transitioning from passive, reactive approaches to proactive techniques aimed at misleading, deterring, and detecting adversaries. Cyber deception, a contemporary manifestation of traditional military deception, has garnered substantial recognition as an effective proactive defense strategy [1]. By implementing decoy systems, honeytokens, and other synthetic artifacts, defenders can establish a misleading environment that entices attackers, depletes their resources, and yields critical intelligence regarding their tactics, techniques, and procedures (TTPs) [2].

This study frames synthetic cyber deception as a detector-mediated decision problem in which attacker behavior is shaped by the information produced by sensing and monitoring systems. In the proposed formulation, the attacker’s observations are treated as evidence generated by detectors (e.g., intrusion detection or monitoring pipelines) with explicit operating characteristics such as false-positive behavior on real assets and detectability behavior on decoys. This perspective enables cyber deception to be analyzed using formal models that connect detector outputs, uncertainty in target authenticity, and strategic attacker responses under defender commitment.

Within this framing, the study uses a game-theoretic structure to compare a baseline transparent security setting to a corresponding deceptive setting under consistent assumptions, thereby enabling the effect of decoys to be interpreted relative to a non-deceptive reference. The resulting evaluation interface emphasizes utility-based measures that remain tied to observable detector outcomes and deployment costs, rather than relying on qualitative claims about deception effectiveness. The study further supports its theoretical statements with reproducible computational validations that are aligned to the assumptions stated for each theorem, including explicit equilibrium computation when required by the value-comparison results.

Notwithstanding its theoretical allure, the practical implementation of cyber deception is frequently obstructed by the challenges associated with assessing its efficacy. Conventional security measures, including the quantity of alerts or thwarted assaults, are inadequate for assessing the efficacy of deception. The efficacy of a deception approach beyond mere assault prevention involves influencing the attacker’s decision-making, heightening their uncertainty, and finally imposing costs that exceed their prospective benefits. This complex interplay of actions and reactions is difficult to capture and quantify, especially in the absence of large-scale, real-world datasets of attacker behavior in deceptive environments [3].

This study presents a method for assessing cyber deception methods grounded in game theory principles. Game theory offers a mathematical framework for examining strategic interactions among rational decision-makers, rendering it an optimal tool for simulating the adversarial dynamics of cybersecurity [4]. We formulate a game-theoretic model that encapsulates the fundamental aspects of cyber deception, encompassing the defender’s utilization of synthetic decoys, the attacker’s efforts to differentiate genuine assets from decoys, and the intrinsic uncertainty and incomplete information that define these interactions. The contributions of this study were the results of experiments that have been suggested in previous work in the literature:

* Standardized, reusable baseline for evaluating cyber deception: The study frames cyber deception evaluation around a fixed, reproducible comparison between an otherwise matched no-deception baseline and a deception-enabled setting. The contribution is not the general idea of using a baseline, but the study’s formalization of this comparison as a repeatable evaluation protocol intended to make results comparable across the heterogeneous deception mechanisms, attacker models, and cost regimes that are an explicit response to the well-documented fragmentation of deception evaluation in the literature [1].
* Baseline-referenced reporting metrics for comparable deception claims (VoD and PoT): The study contributes two explicitly defined, equilibrium-grounded reporting measures, value of deception (VoD) and price of transparency (PoT), that are constructed to be interpreted relative to a matched no-deception baseline, rather than as standalone payoff numbers. This enables cross-setting comparison of deception benefit and transparency cost under differing attacker mixtures, decoy costs, and observability conditions, thereby moving beyond the common practice of reporting isolated “defender utility improved” results that are difficult to compare across models and scenarios [5,6,7,8].
* Formal bounds and break-even conditions that delimit when deception cannot pay off: The study derives explicit, assumption-scoped theoretical results stated as theorems and corollaries that bound the achievable benefit of deception and identify “break-even” regimes in which deception becomes ineffective. These results provide checkable analytical statements showing how deception value must diminish or vanish as key factors worsen (for example, rising decoy costs, increasing attacker discernment, or increasing transparency), thereby clarifying where deception is defensible as a strategy and where it is not within the model class [9].
* Algorithmic structure for heterogeneous decoy allocation with defensible performance claims: The study formulates a heterogeneous decoy-allocation design problem that goes beyond uniform “place decoys everywhere” settings by allowing decoys to differ in cost and effectiveness. Within this formulation, the study identifies structural properties that can be exploited algorithmically, develops scalable allocation rules (including greedy-style selection), and supports them with analytically stated performance properties (and benchmarking against optimal solutions on tractable instances) [10,11,12].
* An uncertainty leakage interpretation layer that explains why deception value changes: The study adds an information-theoretic lens based on attacker uncertainty (conditional entropy) and information leakage to interpret the game-theoretic results, so that shifts in equilibrium value under different transparency and detectability regimes are explained mechanistically through “how much the attacker can infer” rather than reported only as changes in utility [13].
* Robustness analysis under bounded rationality, tied directly to the evaluation metrics: The study strengthens the credibility of its conclusions by moving beyond the assumption of perfectly optimizing attackers and incorporating a bounded-rationality response model (e.g., a quantal-response formulation). The analysis treats attacker rationality as a sensitivity parameter and shows how VoD/PoT and the recommended decoy level change systematically as rationality and attacker-type composition vary. This positions bounded rationality as a structured robustness test of the proposed evaluation framework, rather than as an extension [14,15].
* Reproducible, data-independent benchmarking protocol as a supporting contribution: The study provides a controlled simulation and benchmarking workflow that enables systematic sensitivity analysis across attacker mixtures, decoy costs, and observability regimes when real-world deception datasets are unavailable, incomplete, or difficult to share. A reproducible evaluation artifact is aligned with the paper’s theoretical quantities (equilibrium utilities and the proposed metrics), rather than as a substitute for empirical validation; thus, addressing a widely recognized obstacle in cyber-deception research, namely the difficulty of obtaining standardized datasets and comparable evaluation evidence [16].

The modeling backbone of this study is intentionally aligned with the canonical defender–attacker leader–follower structure used in Stackelberg security games, in which the defender commits to a strategy and the attacker responds given that commitment [17,18]. In this sense, this study contributes a set of evaluation and mechanism-linking constructs that build on this foundation while changing how deception is defined, compared, and interpreted across settings.

First, the study formalizes deception evaluation as an explicit paired-game interface consisting of a transparent baseline security game and a deception-enabled counterpart that are matched in targets, valuations, and accounting conventions, differing only in the availability and structure of deceptive artifacts [1,19]. This pairing provides a controlled baseline-referenced comparison that is distinct from common practice in deception modeling, where deception performance is frequently reported in a single model instance without a systematically matched non-deceptive comparator [20,21].

Second, the study introduces two equilibrium-grounded reporting functionals—the value of deception and the price of transparency—defined directly from equilibrium utilities of the paired games [22]. The conceptual innovation is that these measures are constructed as baseline-referenced objects intended to support cross-scenario comparability under consistent equilibrium selection and cost accounting, rather than as model-specific payoff summaries that are difficult to interpret across different deception mechanisms, attacker populations, and observability regimes [23].

Third, the framework embeds deception mechanisms into detector-mediated and learning-mediated information structures and links these information structures to equilibrium outcomes through explicit, computable decision rules and regime characterizations, including cost- and transparency-driven conditions under which deception becomes ineffective [24]. This mechanism-level emphasis treats deception not only as an expanded defender action set, but as an information-shaping process whose value depends on observability, detectability, and attacker inference [25].

Finally, the study links equilibrium outcomes to a complementary information-disclosure interpretation, providing a mechanism-level explanation for how and why deception value degrades as transparency increases, rather than reporting utility changes alone [26,27]. Overall, the contribution is best understood as an integrative advance over existing Stackelberg security game and deception-based attacker–defender models: it retains the established leader–follower equilibrium backbone while contributing a baseline-referenced paired-game evaluation interface, equilibrium-derived reporting measures, and an information-disclosure linkage intended to be reusable across deception mechanisms and attacker response models [28].

### Synopsis

The conceptual novelty of this study lies not in proposing a wholly new Stackelberg game class, but in establishing a unified evaluation architecture for cyber deception within an existing leader–follower foundation. Specifically, the study formalizes deception assessment through a paired-game interface that couples a transparent security game with a matched deceptive security game under identical targets, payoff conventions, attacker action space, and equilibrium selection, thereby making deception value a baseline-referenced strategic object rather than an isolated utility result. Within this interface, the study introduces the value of deception and the price of transparency as equilibrium-derived comparative measures designed to support cross-setting comparability under consistent cost accounting and attacker response assumptions. The study further contributes a theorem-level regime characterization of deception performance, including explicit break-even conditions, diminishing-returns behavior, upper bounds, and ineffective-deception regimes, and it links these equilibrium outcomes to an information-disclosure interpretation that explains how transparency erodes deception value. Taken together, these contributions position the work as an integrative advance: the novelty resides in the construction of a reusable, baseline-comparable, theorem-supported framework that unifies evaluation, interpretation, and extension of cyber deception models across multiple attacker and observability settings.

This paper is organized as follows. Section 2 provides a brief review of the relevant literature on cyber deception and game theory in cybersecurity. Section 3 presents our game-theoretic framework in detail. Section 4 describes the extensions to sophisticated game models. Section 5 analyzes heterogeneous deception games. Section 6 discusses computational complexity. Section 7 presents the information-theoretic analysis. Section 8 introduces the VoD framework. Section 9 presents and discusses implications, future research directions, and concludes the study.

## 2. Background

Cybersecurity and the contemporary industrial landscape are intricately linked, as Industry 4.0 though to Industry 6.0 increasingly rely on digitization, connection, and automation, all of which broaden the cyber-attack surface. No modern industrial entity can operate securely or dependably without cybersecurity [29,30]. Effective cybersecurity has emerged as a fundamental component of contemporary existence, essential for defense, key infrastructure, and commerce [31,32]. This is primarily attributable to the catastrophic outcomes resulting from inadequate cyber defenses. Recent instances in critical infrastructure include the 2021 Colonial Pipeline breach, which resulted in a six-day shutdown and an approximate increase of four cents in gas prices across 18 states. Additionally, between 2009 and 2017, 133.8 million patients in the United States had their protected health information compromised due to the hacking or IT-related incidents. Therefore, it is of paramount importance that cybersecurity is regarded with seriousness and implemented effectively [31,33,34].

Cybersecurity is inherently interdisciplinary [35,36] and enhancing one’s cybersecurity posture can manifest in several ways [37,38]; however, a critical aspect to consider is cyber decision-making. Cyber defenders universally possess limited resources, although improving cybersecurity typically entails certain expenses. An example of a prevalent cyber protection tool is the intrusion detection system (IDS), which analyzes host or network records to detect anomalous activities [39,40].

These activities inherently incur costs and consume resources (e.g., allocating domain experts to assess IDS alarms) that could be utilized for alternative purposes [41,42]. Consequently, an essential trade-off in cybersecurity decision-making emerges: What level of investment in cybersecurity is warranted considering the advantages of enhanced security against the expenditure of system resources? Addressing this trade-off is a critical issue in cybersecurity, and it is evident that improvised decision-making is inadequate, as any mistakes could incur significant costs [43,44,45].

The application of game theory, which examines the interactions among decision-makers, has been suggested to address this issue. Game theory provides two notable advantages: firstly, its solutions are mathematically optimal, rendering them disciplined and rigorous. Secondly, it expressly addresses the strategic interactions among numerous parties, indicating that the attacker’s reactions to any specific defensive policy are considered. Both traits are essential for disciplined cybersecurity decision-making to achieve an optimal balance between security and efficiency.

Cyber deception under uncertainty examines how information leakage affects attacker–defender outcomes [46]. This is especially relevant in human–AI collaborative manufacturing environments where predictive maintenance and collaborative robots require strong protection [47]. Our research is situated at the intersection of three key domains: cyber deception, game theory in cybersecurity, and attacker modeling. This section provides a comprehensive review of the foundational and recent literature in these areas, establishing the context and identifying the research gap that our proposed framework addresses.

### 2.1. Cyber Deception Techniques and Taxonomies

**Figure 1:**
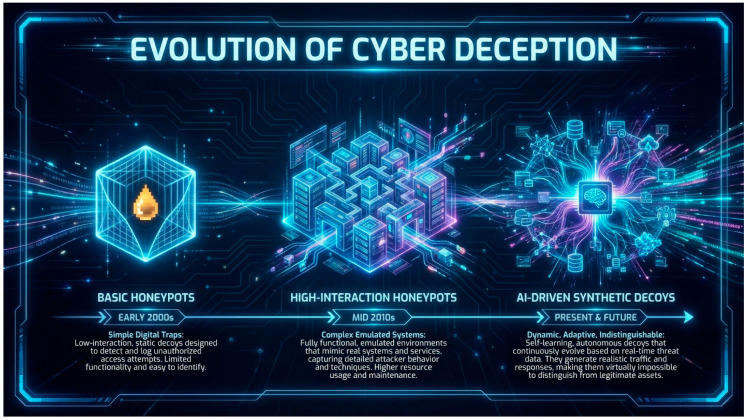
Evolution of deception tactics.

Cyber deception is a proactive defense strategy designed to deceive enemies by altering their perspective of the cyber landscape. The objective is to impose expenses on assailants, deplete their resources, and collect intelligence on their TTPs [48]. The literature defines a diverse range of deception tactics (see Figure 1) that can be generally classified as follows:

- Honeypots: These are decoy systems intended for probing and assault. They vary from low-interaction honeypots that simulate basic services to high-interaction honeypots that offer a comprehensive, monitored environment for attackers. Game-theoretic models have been devised to enhance honeypot deployment, taking into account variables such as attacker probing and the utilization of attack graphs [49,50,51,52].
- Honeytokens: These are deceptive digital artifacts (e.g., fake credentials, API keys, files) that trigger an alert when accessed, serving as high-fidelity intrusion indicators [53,54].
- Moving target defense (MTD): MTD is a proactive defense strategy that dynamically shifts the attack surface (e.g., by changing IP addresses or randomizing memory layouts) to increase uncertainty for attackers. Game theory has been instrumental in analyzing MTD, with models exploring the trade-offs between the security benefits and the operational costs of reconfiguration [55].

Numerous taxonomies have been suggested to organize the extensive array of deception techniques. Pawlick, Colbert, and Zhu’s game-theoretic taxonomy [19] offers a systematic classification grounded in fundamental game-theoretic principles, facilitating comprehension of the strategic intent behind various misleading behaviors.

### 2.2. Game-Theoretic Models for Cybersecurity

Game theory provides a robust mathematical framework for examining strategic interactions in cybersecurity [56]. Signaling games are particularly effective for modeling deception under information asymmetry, where the defender can send signals to influence attacker beliefs and actions. Similarly, GPT-based AI systems, capable of processing multiple languages, can interpret subtle patterns in complex data, enabling adaptive strategies and more informed decision-making under uncertainty. Diverse categories of games have been utilized to simulate various facets of the attacker–defender conflict, as follows:

- Stackelberg security games: These leader–follower models, where the defender commits to a defensive strategy first, are highly applicable to security domains where defensive postures are observable. They have been effectively implemented in practical solutions for infrastructure security. Nonetheless, determining the optimal strategy in these games is frequently NP-hard [57], which has spurred the development of efficient algorithms like the decomposed optimal Bayesian Stackelberg solver (DOBSS) [58].
- Signaling games: These games are optimal for simulating deception in conditions of information asymmetry. The defender (sender) can transmit a signal to the attacker (receiver) to affect their beliefs and behaviors. Pawlick and Zhu’s key work on signaling games with evidence establishes a formal framework for examining leaky deception, when the attacker might potentially discern the deception with a certain probability [59].
- Dynamic and repeated games: Cyber conflicts are seldom isolated incidents. Dynamic and repetitive games represent the long-term, changing interactions between attackers and defenders [60,61,62]. These models integrate learning and adaptation, wherein players modify their strategy based on historical gameplay [63]. The FlipIt game exemplifies the continuous contest for resource management and has been utilized to examine defenses against advanced persistent threats (APTs) [64].
- Information design and Bayesian persuasion: This current research investigates how a defense can strategically construct an information disclosure mechanism to influence an attacker to do activities advantageous to the defender [65]. This approach offers a robust instrument for examining deceit as a method of strategic information disclosure.

### 2.3. Prior Work on Value of Deception

The notion of quantifying the “value” or efficacy of deception has been addressed in previous literature. Zhu et al. [66] notably proposed the concept of “value of deception” within the framework of deceptive routing games, whereas extensive surveys on defensive deception have examined VoD-type metrics as evaluative criteria [16]. Our research extends these foundations by offering precise operationalization of VoD inside a paired-game framework—transparent security game (low-interaction) vs. deceptive security game (DSG)—facilitating direct return-on-investment (ROI) comparability across various deception scenarios. The primary contrast is such that we see VoD not only as a conceptual metric but as a formal interface for standardized deception assessment, supported by clear theoretical limits.

### 2.4. Attacker Modeling and Bounded Rationality

A critical component of any security game model is the model of the attacker. While the assumption of perfect rationality is a useful starting point, it is often unrealistic. The field has seen a growing interest in modeling bounded rationality, which acknowledges that real-world attackers may have cognitive limitations, biases, and incomplete information.

- Quantal response equilibrium (QRE): QRE is a solution concept that relaxes the assumption of perfect rationality by allowing players to make mistakes with a certain probability [14]. The probability of choosing a suboptimal action is inversely related to the expected utility loss. QRE has been shown to provide a better fit for real-world security data than traditional equilibrium concepts [67].
- Nested quantal response (NQR): This is an extension of the QRE model that captures correlations in attacker choices, providing a more scalable and accurate model of adversary behavior [68].
- Learning-based models: Researchers are increasingly using machine learning techniques, such as reinforcement learning, to model adaptive adversaries who learn their strategies over time [69,70]. These models can be trained on data from real or simulated interactions to capture the complex decision-making processes of human attackers.

**Figure 2:**
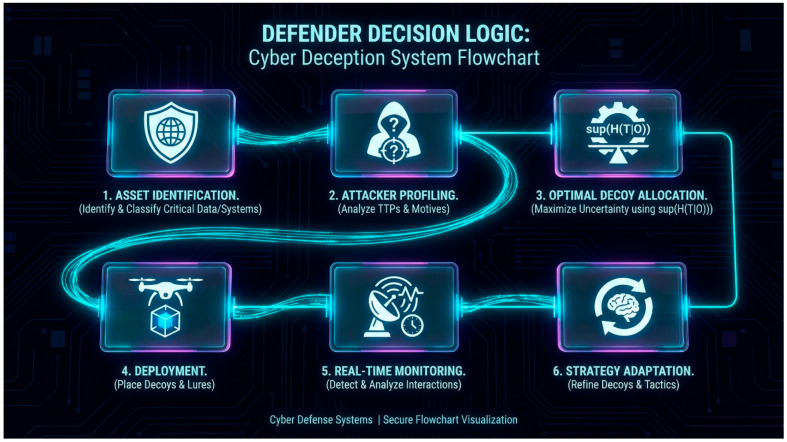
Defender decision logic.

Despite significant progress in these areas, evaluating cyber deception remains a challenge, one that is often hindered by the lack of relevant datasets. Much of the existing work either focuses on a specific type of game, assumes perfect rationality, or lacks a formal connection to the evaluation of deception effectiveness (see Figure 2). Our work aims to bridge this gap by developing a unified Bayesian Stackelberg game framework that can be extended with more sophisticated models like signaling and dynamic games; adapting and operationalizing VoD and PoT as a standardized paired-game evaluation interface; building on prior conceptual work while providing formal bounds and characterization results; providing a formal methodology for evaluating deception strategies based on clear, game-theoretic metrics; analyzing the computational complexity of our proposed framework and discussing scalable solution approaches; and introducing an information-theoretic bridge (“deception capacity”) to quantify residual attacker uncertainty.

## 3. Formal Game-Theoretic Framework

In this section, the game-theoretic framework for analyzing synthetic cyber deception is formally defined. The interaction is modeled as a Bayesian Stackelberg game, which captures the essential characteristics of the problem: a leader–follower dynamic, where the defender commits to a strategy first, and incomplete information, where the defender is uncertain about the attacker’s type [16]. Integrating game-theoretic deception strategies with service-oriented platforms allows for adaptive defense mechanisms that account for dynamic attacker behavior and system uncertainties.

### 3.1. Formal Problem Statement

**Figure 3:**
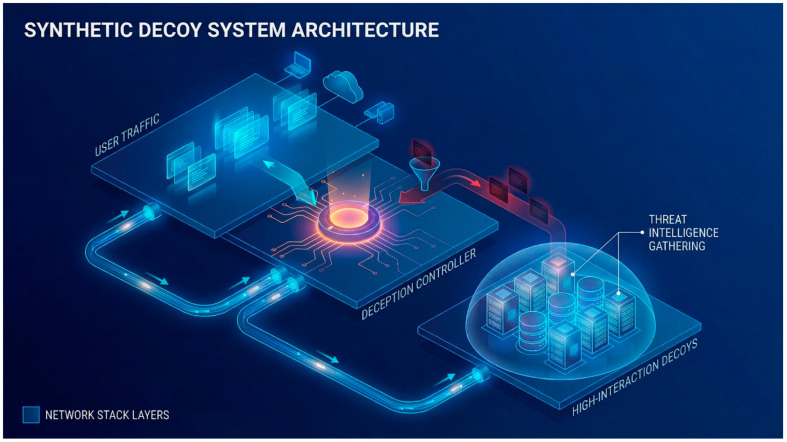
Synthetic decoy system architecture.

The defender’s problem is to find an optimal deception strategy that maximizes their expected utility, given a set of real assets to protect and a population of strategic attackers with unknown types. The deception strategy involves deploying a certain number of synthetic decoys (see Figure 3). The defender must balance the cost of deploying and maintaining these decoys against the benefits of deceiving attackers, which include wasting attacker resources, gathering intelligence, and preventing attacks on real assets [8].

### 3.2. The Bayesian Stackelberg Game Model

The model, which is called the synthetic deception game (SDG), is defined by following the tuple [71]:

(1) Γ=⟨P,T,Θ,p,A,S,U⟩

where we find the following:

- Players (P): The game consists of two players, a defender (D) and an attacker (A). P={D,A}.
- Targets (T): There is a set of N targets, partitioned into a set of real assets $T_{R}$ and a set of synthetic decoys $T_{S}$. $|T_{R}|=N_{R}$ and $|T_{S}|=N_{S}$, with $N=N_{R}+N_{S}$.
- Attacker types (Θ): The attacker has a private type θ∈Θ, where Θ is a finite set of possible attacker types. An attacker’s type encapsulates private information, such as skill, resources, and motivations. For example, Θ={naive,intermediate,advanced}.
- Prior beliefs (p): The defender has a prior belief over the attacker’s type, which is a probability distribution p(θ) for each θ∈Θ, such that $\sum_{\theta \in \Theta }p(\theta )=1$.
- Action spaces (A, S): The defender’s strategy space S is the set of all possible decoy deployment strategies. A pure strategy for the defender is to choose the number of decoys $n_{S}\in \{0,1,\ldots ,N_{max}-N_{R}\}$ to deploy, where $N_{max}$ is the maximum number of possible targets. The defender commits to a strategy s∈S. The attacker’s action space A is the set of all possible targets to attack. $A=T_{R}\cup T_{S}$. The attacker chooses an action a∈A after observing the defender’s strategy.
- Utility functions (U): The utility functions $U_{D}$ and $U_{A}$ define the payoffs for the defender and the attacker, respectively.

#### 3.2.1. Defender’s Utility

The defender’s utility $U_{D}(s,a)$ depends on the chosen strategy s (number of decoys) and the attacker’s action a:

(2) $$U_{D}(s,a)=R_{D}(a)-C_{D}(s)$$

where we find the following:

- $C_{D}(s)$ is the cost of deploying the strategy s. A linear cost function is assumed: $C_{D}(n_{S})=c_{S}\cdot n_{S}$, where $c_{S}$ is the cost per decoy.
- $R_{D}(a)$ is the reward (or loss) to the defender based on the attacker’s action:
  If $a\in T_{S}$ (attacker attacks a decoy), $R_{D}(a)=B_{D}$, where $B_{D}$ is the benefit of detecting an attack (e.g., intelligence gain).
  If $a\in T_{R}$ (attacker attacks a real asset), $R_{D}(a)=-L_{R}$, where $L_{R}$ is the loss incurred from a compromised real asset.

#### 3.2.2. Attacker’s Utility

The attacker’s utility $U_{A}(a,\theta )$ depends on the action a and the type θ:

- If $a\in T_{S}$, $U_{A}(a,\theta )=-C_{A}(\theta )$, where $C_{A}(\theta )$ is the cost to the attacker of type θ for being deceived (e.g., wasted resources, exposure).
- If $a\in T_{R}$, $U_{A}(a,\theta )=R_{A}(\theta )$, where $R_{A}(\theta )$ is the reward to the attacker of type θ for a successful attack.
- The study adopts the following cost-accounting convention. The quantity $R_{A}(\theta )$ is treated as the attacker’s net payoff from successfully compromising a real target, with any target-independent execution cost already absorbed into that term. The parameter $C_{A}\left(\theta \right)$ is reserved exclusively for the incremental loss attributable to deception outcomes—namely, the additional operational penalty incurred when a decoy is engaged (e.g., wasted effort, increased exposure, tool attrition, or mission setback). Under this convention, the attacker’s utility subtracts $C_{A}(\theta )$ only in the decoy outcome, thereby preventing double counting of a universal per-attack cost. If an alternative convention is preferred—where $R_{A}(\theta )$ denotes a gross success reward and a universal execution cost is modeled explicitly—the equilibrium statements and proofs remain unchanged after a notational reparameterization that introduces a per-attack cost term and correspondingly redefines $R_{A}(\theta )$ to preserve identical net payoffs.

### 3.3. Equilibrium Analysis

The strong Stackelberg equilibrium (SSE) is used as the solution concept [12]. In an SSE, the defender chooses a strategy $s^{*}$ that maximizes the defender’s expected utility, anticipating the attacker’s best response. The attacker, in turn, chooses an action that maximizes the attacker’s utility, breaking ties in favor of the defender [72,73].

#### 3.3.1. Attacker’s Best Response

For a given defensive strategy s (i.e., a given number of decoys $n_{S}$), an attacker of type θ will choose an action $a^{*}$ that maximizes the attacker’s expected utility. The attacker’s decision depends on the attacker’s ability to distinguish real assets from decoys. Let $P(a\in T_{R}|s,\theta )$ be the probability that an attacker of type θ correctly identifies and attacks a real asset, given the defender’s strategy s. This probability is a function of the attacker’s discernment d(θ) and the ratio of real assets to total targets [74]. Thus, the attacker’s expected utility for attacking is as follows:

(3) $$E[U_{A}(s,\theta )]\;=\;P(a\in T_{R}|s,\theta )\cdot R_{A}(\theta )\;+\;(1-P(a\in T_{R}|s,\theta ))\cdot (-C_{A}(\theta ))$$

The attacker will attack if $E[U_{A}(s,\theta )]>0$.

#### 3.3.2. Defender’s Optimal Strategy

The defender’s problem is to choose a strategy $s^{*}$ that maximizes the defender’s expected utility [75], $E[U_{D}(s)]$, which is the sum of the defender’s utilities against each attacker type, weighted by the prior probabilities p(θ):

(4) $$s^{*}\;=\;arg\;\underset{s\in S}{max}\;E[U_{D}(s)]\;=\;arg\;\underset{s\in S}{max}\sum_{\theta \in \Theta }p(\theta )\cdot U_{D}(s,a^{*}(s,\theta ))$$

where $a^{*}(s,\theta )$ is the best response of an attacker of type θ to the defender’s strategy s.

### 3.4. Theorem 1: Existence of Optimal Strategy

**Theorem** **1.** *In SDG with a finite number of attacker types and a finite number of pure strategies for the defender, there always exists an optimal pure strategy for the defender.*

**Proof.**  Finite strategy space: The defender’s set of pure strategies S is the set of possible numbers of decoys to deploy, $n_{S}\in \{0,1,\ldots ,N_{max}-N_{R}\}$. This is a finite set. □

Well-defined utility: For any given defender strategy s∈S, the attacker’s best response is well-defined. As the attacker’s utility function $U_{A}(a,\theta )$ is defined for all actions and types, an attacker of type θ will choose an action $a^{*}(s,\theta )$ that maximizes the attacker’s utility. If there are multiple such actions, the tie-breaking rule (in favor of the defender) ensures a unique best response. For each θ∈Θ and each s∈S, because A is finite and $U_{A}(a,\theta )$ is real-valued, the best-response set, $BR(s,\theta )=arg\;{max}_{a\in A}U_{A}(a,\theta )$, is non-empty. Under the strong/optimistic convention, $a^{*}(s,\theta )$ is selected from BR(s,θ) to maximize the defender’s payoff.

Computable expected utility: For any defender strategy s∈S, the defender’s expected utility $E[U_{D}(s)]$ can be computed by summing over the finite set of attacker types Θ, as follows:

(5) $$E[U_{D}(s)]\;=\;\sum_{\theta \in \Theta }p(\theta )\cdot U_{D}(s,a^{*}(s,\theta ))$$

As Θ is finite and $U_{D}$ is well-defined for all outcomes, $E[U_{D}(s)]$ is a real-valued number for each s∈S.

Existence of maximum: Because S is finite, $E[U_{D}(s)]$ attains a maximum on S. As S is a finite set and $E[U_{D}(s)]$ is a real-valued function on S, there must exist at least one strategy $s^{*}\in S$ such that $E[U_{D}(s^{*})]\geq E[U_{D}(s)]$ for all s∈S. This $s^{*}$ is an optimal pure strategy for the defender.

This theorem provides a formal well-posedness guarantee for the SDG by establishing that the defender’s discrete decoy-deployment decision admits an optimal commitment under finite attacker heterogeneity. In the context of synthetic cyber deception, this result supplies the foundational justification for computing strong Stackelberg solutions over integer decoy counts without relying on implicit existence assumptions.

#### Validation of Theorem 1

**Figure 4:**
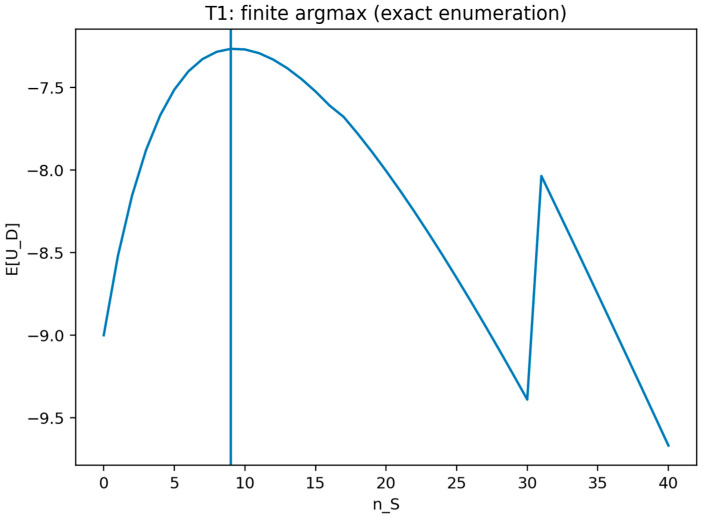
Exact finite strategy maximizer of defender utility in the SDG.

Theorem 1 was validated (see Figure 4) by treating the defender strategy as an integer choice over all feasible decoy counts and computing the defender objective for every feasible value in that finite set. The certificate records the objective profile and the maximizing decoy count. The validation is marked as passing when the reported maximizer attains the largest objective value in the enumeration.

This figure plots the defender objective value across the finite set of feasible decoy counts and marks the maximizing choice. It visually confirms that the optimum is attained within the finite pure-strategy set and by indicating the location of at least one maximizer.

## 4. Extensions to Sophisticated Game Models

The basic SDG model provides a foundation for analyzing cyber deception. However, real-world scenarios often involve more complex dynamics. This section extends the framework to incorporate signaling games, dynamic games, and bounded rationality.

Each extension retains the same leader–follower commitment structure used in the SDG. The defender (leader) commits first to the extension-specific decision variables (e.g., quality–quantity parameters in signaling-with-evidence, rotation timing in the dynamic model, or the committed decoy-count policy under bounded rationality). The attacker (follower) then observes the defender’s commitment and any extension-specific observations (e.g., evidence from a detector, elapsed time since rotation, or expected utility scores inside the logit rule) and selects an action that maximizes the attacker’s objective under the corresponding behavioral model. Defender utility is always evaluated as the defender’s expected utility under the induced attacker response, and all added extension parameters are defined so that (i) the attacker’s decision rule is explicit, (ii) the defender’s optimization target is explicit, and (iii) the mapping from defender decisions to expected utilities is fully specified by stated probabilities and costs.

### 4.1. Signaling Games for Leaky Deception

The basic SDG model assumes that the attacker only observes the number of targets, not any specific signals about their authenticity. A more realistic scenario can be modeled using a signaling game, where the defender can send signals to the attacker, and the attacker’s ability to detect deception is explicitly modeled.

Following the work of Pawlick et al. [19,59], the model can be extended to a signaling game with evidence. In this extension, the defender’s decoys are not perfect; they are “leaky” and can be detected with a certain probability. The defender’s strategy now includes not only the number of decoys but also the quality of the decoys, which affects their detectability [76].

#### 4.1.1. Theorem 2: Budgeted Quality–Quantity Tradeoff Under Leaky Deception

Let $N_{R}$ denote the number of real assets and let the defender deploy n decoys, so that the total number of targets is $N_{R}+n$. A detector emits evidence e∈{0,1} for a probed target.

Detector semantics and attack timing: Evidence is binary, e∈{0,1}. The value e=1 denotes an alarm/flag produced by the detector, whereas e=0 denotes a “no-alarm” outcome. The detector operating characteristics are defined as follows: β=Pr(e=1∣real) is the false-positive rate on real assets and δ(q)=Pr(e=1∣decoy of quality q) is the decoy detectability function, so Pr(e=0∣decoy,q)=1−δ(q) is the corresponding false-negative probability on decoys. The attacker observes the realized evidence e and then decides whether to attack; the model does not restrict attacks to e=0. However, the defender’s deterrence design objective in Theorem 2 is stated as deterrence after e=0 because e=0 corresponds to the operationally important “no-alarm” regime in which an attacker is not immediately warned away by detector output, and deterrence must therefore arise from posterior uncertainty rather than from explicit alarm signaling.

Let p=Pr(real∣e) denote the attacker’s posterior belief that the probed target is real after observing evidence e. Under the stated payoff convention, attacking yields expected utility $p\cdot R_{A}(\theta )-(1-p)\cdot C_{A}\left(\theta \right)$. The attacker attacks if and only if this quantity is nonnegative, which is equivalent to $p\geq \frac{C_{A}\left(\theta \right)}{R_{A}(\theta )+C_{A}\left(\theta \right)}$.

The detector’s false-positive rate is β∈(0,1), where Pr(e=1∣real)=β. A decoy has “quality” q≥0, and its detectability is α(q)∈(0,1), where Pr(e=1∣decoy)=α(q), with $\alpha^{\prime }(q)<0$ and α continuous. The per-decoy cost is c(q), with $c^{\prime }(q)>0$, c continuous, and the deception budget is B>0, so n c(q)≤B. For an attacker type θ, we define the attack threshold as follows:

(6) $$\tau (\theta )=\frac{C_{A}(\theta )}{R_{A}(\theta )+C_{A}(\theta )}\in (0,1),$$

Equation (6) defines this quantity as $\tau_{\theta }$ and thus provides the posterior-belief cutoff separating “attack” from “decline to attack” for each attacker type θ. Therefore, the attacker attacks a target after observing evidence e if and only if the posterior probability that the target is real satisfies Pr(real∣e)≥τ(θ).

**Theorem** **2.** *Fix any attacker type θ. Suppose the defender’s design objective in the signaling-with-evidence extension is to choose (n,q) to make the attacker decline to attack after observing e=0 (the “no-alarm” signal), while respecting the budget n c(q)≤B. If the feasibility conditions B>0 and β<τ(θ) hold, then the set of feasible designs (n,q) is non-empty. Moreover, for any two detectors with the same β and decoy detectability functions $\alpha_{1}(q)$ and $\alpha_{2}(q)$ such that $\alpha_{2}(q)\geq \alpha_{1}(q)$ for all q (i.e., detector 2 is weakly more likely to flag decoys at every quality level), the minimal required decoy proportion $\frac{n}{N_{R}+n}$ to deter attack after e=0 is weakly larger under detector 2 at every fixed q. Under the budget constraint, this implies that any optimal deterrence-feasible design shifts weakly toward higher quality q and lower quantity n when α(q) increases pointwise (holding β fixed), provided c(q) is strictly increasing.*

**Proof.**  Let $\pi_{R}=\frac{N_{R}}{N_{R}+n}$ and $\pi_{S}=\frac{n}{N_{R}+n}=1-\pi_{R}$. Under Bayes’ rule, the posterior after observing e=0 is as follows:

(7) $$Pr(\mathrm{real}|e=0)=\frac{\pi_{R}(1-\beta )}{\pi_{R}(1-\beta )+\pi_{S}(1-\alpha (q))}.$$

□

Deterrence after e=0 requires Pr(real∣e=0)<τ(θ). This inequality is equivalent to the following:

(8) $$\pi_{R}(1-\beta )\;<\;\tau (\theta )(\pi_{R}(1-\beta )+\pi_{S}(1-\alpha (q))),$$

which rearranges to the following:

(9) $$\frac{\pi_{S}}{\pi_{R}}\;>\;\frac{\left(1-\beta )(1-\tau (\theta )\right)}{\tau (\theta )\;(1-\alpha (q))}.$$

As $\frac{\pi_{S}}{\pi_{R}}=\frac{n}{N_{R}}$, deterrence is equivalent to the following:

(10) $$n\;>\;N_{R}\cdot \frac{\left(1-\beta )(1-\tau (\theta )\right)}{\tau (\theta )\;(1-\alpha (q))}.$$

Define the right-hand side as h(α(q)). Because 1−α(q)∈(0,1), h is strictly increasing in α(q). Therefore, for any fixed q, if $\alpha_{2}(q)\geq \alpha_{1}(q)$, then $h(\alpha_{2}(q))\geq h(\alpha_{1}(q))$, implying the minimal required n (hence the minimal required decoy proportion $\pi_{S}$) is weakly larger under detector 2 at that q. Under the budget n c(q)≤B, if the required n increases at fixed q, feasibility can be restored only by decreasing α(q), which—as $\alpha^{\prime }(q)<0$—requires increasing q. Because c(q) is strictly increasing, raising q tightens the budget and forces n≤B/c(q) downward. Hence the shift is weakly toward higher q and lower n. Non-emptiness under β<τ(θ) follows because h(α(q)) is finite for any q with α(q)<1, and a sufficiently large q makes α(q) sufficiently small to reduce h, while a sufficiently large B permits n to satisfy both constraints. Thus, providing a precise deterrence condition and derives a budget-mediated quality–quantity implication from that condition.

This theorem formalizes the leaky-decoy setting by linking detector operating characteristics and decoy design choices to the defender’s optimal deception posture within a signaling-with-evidence extension of the SDG. In the context of cyber deception engineering, the result yields a principled design interpretation for how detector strength and decoy credibility jointly shape the defender’s optimal quality–quantity tradeoff.

The signaling-with-evidence extension parameterizes decoy “quality” q through two functional relationships: a decoy detectability function α(q) (capturing how easily a decoy is flagged/recognized under the detector’s evidence process) and a per-decoy cost function c(q) (capturing the marginal expense of deploying higher-fidelity decoys). These are two qualitative assumptions that reflect standard operational regularities in deception engineering. First, α(q) is assumed to be monotone in q, meaning that increases in quality shift detectability in a single direction (in the realism interpretation used in this section, higher q corresponds to higher fidelity and therefore weakly lower detectability). This monotonicity is intended as an order property rather than a commitment to a particular parametric curve shape; it covers smooth forms (e.g., saturating or S-shaped behavior) as well as piecewise-smooth forms that may arise when quality improvements remove salient fingerprints. Second, c(q) is assumed to be strictly increasing in q, and is taken to be convex when the analysis requires an increasing marginal cost of realism. This captures the empirical engineering pattern that low-fidelity decoys are cheap to deploy, whereas high-fidelity decoys require greater integration effort, maintenance, and monitoring overhead, so that incremental realism becomes progressively more expensive.

These assumptions are introduced to ensure that (i) quality choices are economically meaningful under a budget and that (ii) the deterrence-feasible set exhibits an interpretable quality–quantity trade-off. Importantly, the core deterrence condition in this model is not tied to a specific smooth functional form: the posterior expression Pr(real∣e) is computed by Bayes’ rule using the detector operating characteristics, and the attacker’s attack decision depends on whether this posterior crosses the type-dependent threshold. Consequently, for any admissible α(q) and c(q) (including non-smooth or threshold-like behavior), the deterrence criterion is evaluated exactly through the posterior inequality; what changes across functional forms is the geometry of the feasible design region and, therefore, which (q,K) pairs satisfy the budget and posterior constraints. When α(q) exhibits threshold effects or other non-smooth behavior, the same posterior-based deterrence inequality continues to define feasibility, but the implied optimal designs naturally become piecewise, with quality choices concentrating at levels that cross or avoid the threshold—an outcome consistent with engineering practice in which eliminating a single fingerprint can produce a discrete jump in adversary discrimination capability [77].

##### Validation of Theorem 2

**Figure 5:**
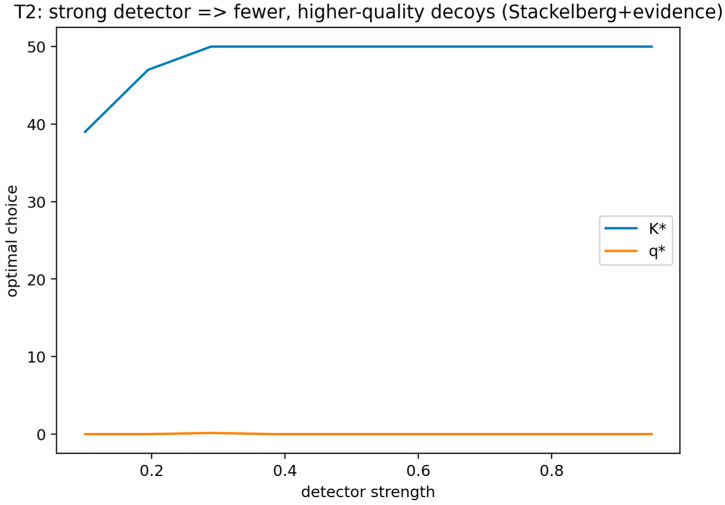
Detector-induced quality–quantity tradeoff under leaky deception.

**Figure 6:**
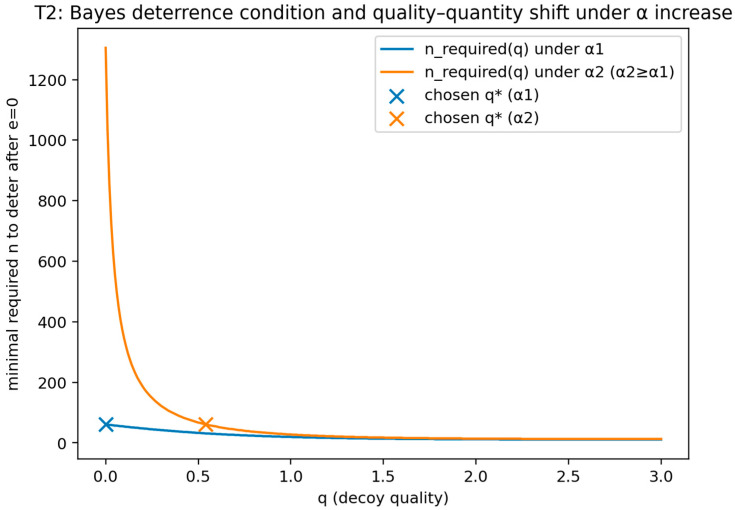
Bayes posterior deterrence condition and budget-feasible quality–quantity region.

Theorem 2 was validated (see Figure 5 and Figure 6) by implementing the signaling-with-evidence model exactly as stated, including binary evidence, a real-target false-positive rate beta, and a quality-dependent decoy detectability function α(q) that decreases with quality, together with a per-decoy cost c(q) that increases with quality and a budget constraint. The validation computes the Bayes posterior Pr(real | e = 0) and verifies the deterrence condition Pr(real | e = 0) < τ(θ).

A second check enforces the theorem’s comparative static by evaluating two detectability functions with pointwise ordering and confirming that, at fixed quality, the minimal required decoy proportion is weakly larger under the more detectable decoy function; under the budget, the deterrence-feasible design is observed to shift toward higher quality and lower quantity.

This figure compares the minimum required decoy proportion or the deterrence-feasible design region under two detectability functions that are pointwise ordered. It shows that, at fixed quality, the minimal required decoy proportion is weakly larger when the detector flags decoys more frequently, and by illustrating the induced shift toward higher quality and lower quantity under a binding budget constraint.

The x-axis reports decoy quality q, which parameterizes decoy detectability through δ(q)=Pr(e=1∣decoy,q) and cost through c(q). The y-axis reports the minimum deterrence-feasible decoy proportion $\rho_{min}(q)$ (equivalently, the minimum decoy count $K_{min}(q)$ at fixed $N_{R}$) required to satisfy the posterior deterrence inequality $Pr(\mathrm{real}|e=0)<\tau_{\theta }$, where the posterior is computed by Bayes’ rule using β=Pr(e=1∣real) and δ(q). “Detector strength” is operationalized via the pointwise ordering of detectability functions (higher δ(q) implies that the detector flags decoys more frequently at the same q). For each q, $\rho_{min}(q)$ is obtained by solving the rearranged deterrence condition (Equation (10)) for the smallest feasible K satisfying the budget and posterior constraints; thus, the plotted curve represents a computed feasibility boundary rather than a fitted regression.

This figure visualizes the Bayes posterior deterrence condition after observing the no-alarm evidence signal e = 0 and shows how the minimum deterrence-feasible decoy count varies with quality. It exhibits the computed posterior-based feasibility boundary that defines the required decoy proportion for deterrence at each quality level under the stated parameters.

### 4.2. Repeated and Dynamic Games for Advanced Persistent Threats

Advanced persistent threats (APTs) are characterized by their long-term, stealthy nature [78]. To model the interaction with APTs, the framework is extended to a dynamic game setting [79]. In a repeated game setting, the defender and attacker interact over multiple rounds [78]. The defender can adapt the deception strategy based on the attacker’s past actions, and the attacker can learn about the defender’s strategy over time [80]. This introduces the possibility of reputation and punishment strategies [81].

The FlipIt game model [82] is particularly relevant here. The control of each asset can be modeled as a separate FlipIt game, where the defender and attacker compete for control. The defender’s deception strategy would then involve deciding which assets to “flip” (i.e., turn into decoys or back into real assets) and when [83].

#### 4.2.1. Theorem 3: Closed-Form Optimal Rotation Period Under APT Learning

Consider a dynamic deception policy in which the defender “rotates” the configuration of assets/decoys periodically with a period Δ>0. Rotation resets the attacker’s knowledge to baseline. Let the attacker’s probability of correctly identifying real assets at elapsed time t∈[0,Δ] after rotation be as follows:

(11) $$\rho (t)=1-e^{-\kappa t},$$

where κ>0 is the attacker learning rate. Let the expected loss rate (per unit time) from successful targeting when knowledge level is ρ be L ρ(t), where L>0. Each rotation incurs cost K>0. The defender minimizes long-run average cost per unit time.

Interpretation of attacker knowledge and learning-rate parameter: The quantity p(t) in Equation (11) represents the attacker’s knowledge level, operationalized as a probability—specifically, the probability that the attacker correctly identifies real assets (or equivalently, avoids decoys) at elapsed time t after the last rotation. The parameter λ is the attacker learning-rate constant, governing how rapidly this probability increases over time. Earlier drafts may denote the same learning-rate parameter by L; for consistency throughout the study, the learning rate is denoted by λ in all dynamic-rotation statements and validations.

**Theorem** **3.** *The long-run average cost of a periodic rotation policy with period Δ is as follows:*

(12) $$J(\Delta )=\frac{K}{\Delta }+\frac{1}{\Delta }\int_{0}^{\Delta }L(1-e^{-\kappa t})\;dt\;=\;\frac{K}{\Delta }+L\left(1-\frac{1-e^{-\kappa \Delta }}{\kappa \Delta }\right)$$

*If $0<K<\frac{L}{\kappa }$, there exists a unique minimizer $\Delta^{*}\in (0,\infty )$ characterized by the first-order condition, as follows:*

(13) $$K=\frac{L}{\kappa }(\;1-e^{-\kappa \Delta^{*}}-\kappa \Delta^{*}e^{-\kappa \Delta^{*}}\;)$$

*If $K\geq \frac{L}{\kappa }$, then J(Δ) is minimized in the limit Δ→∞, and the optimal policy is to not rotate.*

**Proof.**  The expression for J(Δ) follows by direct integration is as follows:

(14) $$\int_{0}^{\Delta }(1-e^{-\kappa t})dt=\Delta -\frac{1-e^{-\kappa \Delta }}{\kappa }$$

□

Differentiating J(Δ) yields the following:

(15) $$J^{\prime }(\Delta )=-\frac{K}{\Delta^{2}}+L\cdot \frac{(1-e^{-\kappa \Delta })-\kappa \Delta e^{-\kappa \Delta }}{\kappa \Delta^{2}}$$

Setting $J^{\prime }(\Delta )=0$ gives the stated first-order condition. Define the following:

(16) $$g(\Delta )=\frac{L}{\kappa }(\;1-e^{-\kappa \Delta }-\kappa \Delta e^{-\kappa \Delta }\;)$$

Then g(0)=0 (by a second-order expansion) and ${lim}_{\Delta \to \infty }g(\Delta )=\frac{L}{\kappa }$. Moreover, we find the following:

(17) $$g^{\prime }\left(\Delta \right)=\frac{L}{\kappa }\cdot \kappa^{2}\Delta e^{-\kappa \Delta }=L\kappa \Delta e^{-\kappa \Delta }>0\;\mathrm{for}\;\mathrm{all}\;\Delta >0,$$

Thus, g is strictly increasing on (0,∞). Therefore, if $0<K<\frac{L}{\kappa }$, there exists a unique $\Delta^{*}$ solving $K=g(\Delta^{*})$. If $K\geq \frac{L}{\kappa }$, no finite Δ solves K=g(Δ), and, since $\frac{K}{\Delta }\to 0$, while the second term approaches L, the infimum is attained as Δ→∞. This establishes existence and uniqueness as stated. The time-and-control interpretation is consistent with FlipIt-style APT modeling where control/knowledge evolves over time and timing decisions are central, thus providing an explicit optimal timing rule (with parameter dependence on κ, K, and L).

This theorem introduces a dynamic structural implication for synthetic deception against APTs by characterizing why optimal decoy management must be policy-driven rather than static, with timing governed by learning and reconfiguration costs. In the SDG extension to repeated/dynamic interaction, the result identifies a concrete policy lever—decoy rotation timing—that is directly actionable for operational deception deployments.

##### Validation of Theorem 3

**Figure 7:**
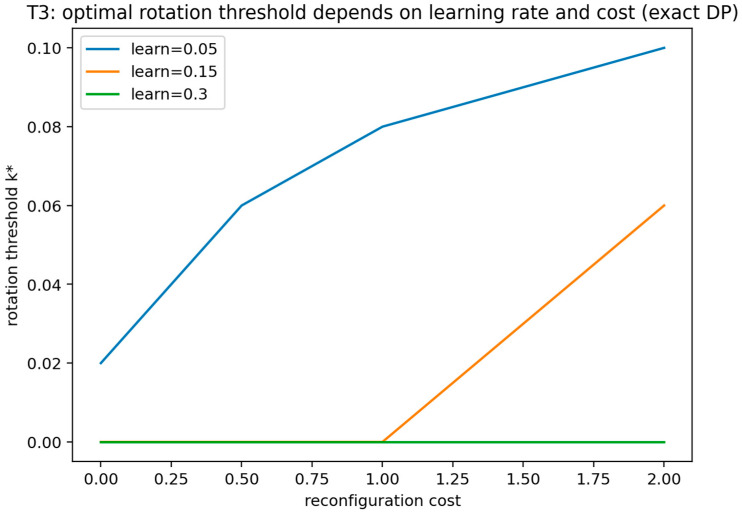
Optimal decoy rotation thresholds in a dynamic deception game.

Theorem 3 was validated (see Figure 7) by instantiating a finite-horizon dynamic deception model in which the defender chooses time-indexed reconfiguration actions and the attacker effectiveness evolves according to an explicit learning-rate parameter, while the defender pays a stated reconfiguration cost. The validation computes an optimal policy over the model’s state space using dynamic programming and records the resulting policy and value function. The validation is marked as passing when the optimal action is not constant over time or state, and when policy changes occur in the directions predicted by the learning-rate and cost parameters used in the instantiated model class.

This figure shows the defender’s computed time-dependent reconfiguration policy or rotation threshold as model parameters vary, including the attacker learning rate and the defender reconfiguration cost. It demonstrates that the optimal deception strategy is not static by showing systematic policy shifts with the learning-rate and cost parameters within the instantiated dynamic model class.

### 4.3. Bounded Rationality and Quantal Response

The assumption of perfect attacker rationality is often unrealistic. To address this, the QRE model is incorporated [84,85]. In a QRE model, players choose their actions stochastically. The probability of choosing a particular action is an increasing function of its expected utility. This means that players are more likely to choose better actions, but they do not always choose the best one. The defender’s expected utility is now calculated by taking the expectation over the attacker’s stochastic choices. The defender’s problem is still to choose a strategy that maximizes this expected utility [76].

#### 4.3.1. Theorem 4: Finite-λ Rationality Bound for Logit QRE in SDG

For a fixed defender commitment s and attacker type θ, let the attacker’s expected utility for action a∈A be $u(a)=E[U_{A}(a,s,\theta )]$. Under logit QRE with rationality $\lambda_{A}\geq 0$, the attacker chooses the following:

(18) $$P_{\lambda_{A}}(a)=\frac{exp(\lambda_{A}u(a))}{\sum_{a^{\prime }\in A}exp(\lambda_{A}u(a^{\prime }))}$$

Let $u^{*}={max}_{a\in A}u(a)$, let $A^{*}=\{a:u(a)=u^{*}\}$, and define the utility gap $\Delta_{min}=u^{*}-{max}_{a\notin A^{*}}u(a)$, with the convention $\Delta_{min}=0$ if $A^{*}=A$.

**Theorem** **4.** *If $\Delta_{min}>0$, then for every $\lambda_{A}\geq 0$, we find the following:*

(19) $$1-\sum_{a\in A^{*}}P_{\lambda_{A}}(a)\;\leq \;(|A|-|A^{*}|)\;e^{-\lambda_{A}\Delta_{min}}$$

Consequently, the probability of selecting a non-best-response action decays to zero at an exponential rate in $\lambda_{A}$, and the induced attacker behavior converges to best-response play. If $\lambda_{A}\to 0$, then $P_{\lambda_{A}}(a)\to 1/|A|$ for all a∈A.

**Proof.**  For any $a\notin A^{*}$, $u(a)\leq u^{*}-\Delta_{min}$. Therefore, we find the following:

(20) $$exp(\lambda_{A}u(a))\leq exp(\lambda_{A}(u^{*}-\Delta_{min}))=exp(\lambda_{A}u^{*})e^{-\lambda_{A}\Delta_{min}}$$

□

Summing over $a\notin A^{*}$ gives the following:

(21) $$\sum_{a\notin A^{*}}exp(\lambda_{A}u(a))\leq (|A|-|A^{*}|)exp(\lambda_{A}u^{*})e^{-\lambda_{A}\Delta_{min}}$$

Additionally, $\sum_{a^{\prime }\in A}exp(\lambda_{A}u(a^{\prime }))\geq \sum_{a^{\prime }\in A^{*}}exp(\lambda_{A}u(a^{\prime }))=|A^{*}|exp(\lambda_{A}u^{*}).$ Hence, we find the following:

(22) $$\begin{array}{cc} 1-\sum_{a\in A^{*}}P_{\lambda_{A}}\left(a\right)= & \frac{\sum_{a\notin A^{*}}exp(\lambda_{A}u(a))}{\sum_{a^{\prime }\in A}exp(\lambda_{A}u(a^{\prime }))}\\ \; & \leq \frac{\left(|A|-|A^{*}|\right)\exp\left(\lambda_{A}u^{*}\right)e^{-\lambda_{A}\Delta_{min}}}{|A^{*}|\exp\left(\lambda_{A}u^{*}\right)}\\ \; & \leq (|A|-|A^{*}|)e^{-\lambda_{A}\Delta_{min}}, \end{array}$$

where the last inequality uses $|A^{*}|\geq 1$. The $\lambda_{A}\to 0$ limit follows because $\exp\left(\lambda_{A}u\left(a\right)\right)\to 1$ for all a, so the denominator tends to ∣A∣. This bound and the logit formulation are standard within QRE; the distinctive element here is the explicit finite-$\lambda_{A}$ deviation bound stated in SDG notation, thus providing explicit quantitative guarantee on bounded rationality effects.

This theorem connects bounded rationality to synthetic deception outcomes by specifying how attacker behavior transitions between uniform exploration and best-response targeting as rationality varies under the QRE model. In the SDG setting, this result provides a defensible bridge between idealized perfect rationality assumptions and empirically plausible attacker decision noise, enabling sensitivity analysis of deception performance across rationality regimes.

##### Validation of Theorem 4

**Figure 8:**
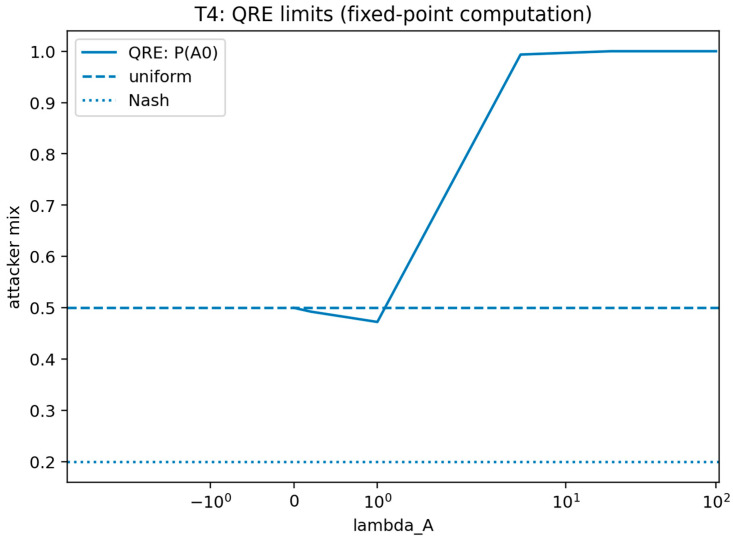
Logit quantal response equilibrium across rationality levels.

**Figure 9:**
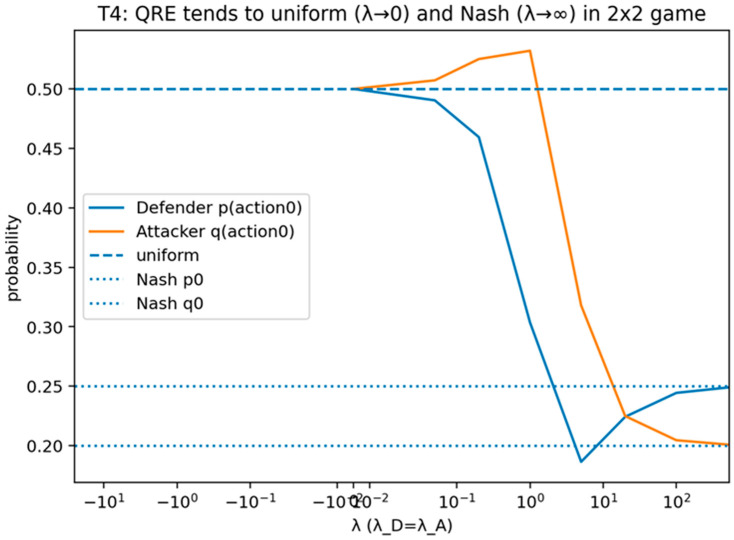
Root-solved QRE fixed-point validation and limiting behavior.

Theorem 4 was validated (see Figure 8 and Figure 9) by computing logit quantal response equilibrium choice probabilities over a sweep of the rationality parameter using an explicit fixed-point solver and recording convergence diagnostics.

For each rationality value λ in Figure 8 and Figure 9, the logit rule induces an attacker mixed strategy $\pi_{\lambda }$ over the finite action set A, where $\pi_{\lambda }(a)$ is the probability assigned to action a as defined in Equation (18). The term “attacker mix” refers to this probability distribution $\pi_{\lambda }$ (either presented as the full vector across actions or summarized by selected components such as $\pi_{\lambda }(a^{*})$ for a best-response action $a^{*}$). Accordingly, the plotted “probability” values represent choice probabilities under bounded rationality rather than empirical frequencies.

The validation compares the computed mixed strategy to the limiting behaviors referenced by the theorem. The validation is marked as passing when the distribution approaches uniform randomization as rationality approaches zero and approaches deterministic best-response behavior as rationality becomes large, within a specified numerical tolerance.

This figure provides a simplified view of the same quantal response limit behavior by plotting action selection probabilities across the rationality sweep. It illustrates the approach to uniform randomization for small rationality values and the approach to deterministic best-response behavior for large rationality values.

Figure 8 shows attacker mixed strategy $\pi_{\lambda }$ under logit QRE as the rationality parameter varies. The y-axis reports $\pi_{\lambda }(a)$, the model-implied probability of selecting each action a under Equation (18), and the x-axis reports the rationality parameter λ. The plot illustrates the limiting behaviors stated in Theorem 4: as λ→0, the distribution approaches uniform randomization, and as λ increases, probability mass concentrates on best-response actions.

This figure reports the fixed-point solution outcomes for logit quantal response equilibrium as the rationality parameter varies and includes convergence diagnostics or reference lines for limiting behavior. It shows convergence toward uniform choice at low rationality and concentration toward best responses at high rationality, with the root-solver output providing the computational certificate.

Figure 9 shows numerical certificate for QRE computation across rationality values. For each rationality level λ, a fixed-point/root solver is used to compute the logit-consistent mixed strategy $\pi_{\lambda }$. The y-axis reports a solver certificate (the residual norm of the fixed-point conditions, together with the associated best-response probability mass $\pi_{\lambda }(a^{*})$) to document that the plotted mixtures satisfy the QRE equations to the stated numerical tolerance. The figure thus distinguishes (i) behavioral concentration effects from (ii) numerical convergence, strengthening the defensibility of the bounded-rationality validation.

## 5. Optimal Allocation in Heterogeneous Deception Games (HDGs)

The basic SDG is extended to allow for K different types of decoys [86]. Each decoy type k has the following:

- A deployment cost $c_{k}$.
- An effectiveness $e_{k}$, which represents the probability that an attacker who interacts with a decoy of type k is detected.
- A quality $q_{k}$, which affects how easily the decoy can be distinguished from a real asset.

The defender’s strategy is a vector $n=(n_{1},n_{2},\ldots ,n_{K})$, where $n_{k}$ is the number of decoys of type k deployed. The total number of decoys is as follows:

(23) $$n_{S}=\sum_{k=1}^{K}n_{k}$$

In the heterogeneous deception game, the defender commits to an allocation vector $K=(K_{1},\ldots ,K_{m})$ specifying the number of decoys deployed of each type, subject to a budget constraint. After observing this commitment, the attacker’s interaction with decoys is modeled at the type level: the attacker selects which decoy-type class to engage with according to a probability vector $\pi =(\pi_{1},\ldots ,\pi_{m})$ (or, in the baseline theorem, π is treated as exogenous and fixed). Conditional on engaging with a decoy of type k, the defender detects the attacker with probability $e_{k}$, yielding detection benefit $B_{D}$ to the defender. Accordingly, the defender’s expected utility contribution from type-k decoys is proportional to $\pi_{k}e_{k}B_{D}K_{k}$, and the defender’s optimization objective is the expected detection benefit minus deployment costs, evaluated under the stated π and $e_{k}$ assumptions.

In this section, “attacker interaction with a decoy” is used as a neutral term encompassing an attack act on a decoy (e.g., probing, an exploitation attempt, or any engagement sufficient to trigger defender telemetry). Accordingly, “interaction” is not distinct from “attack”; it is the decoy-side realization of an attack attempt.

The parameter $e_{k}$ is the conditional detection probability given interaction with a type-k decoy. To avoid redundant parameterization, $\pi_{k}$ is interpreted as the attacker’s probability of selecting the type-k decoy class to engage with (and, under the theorem, $\pi_{k}$ is treated as fixed), so the effective probability of a defender detection event attributable to type k is $\pi_{k}e_{k}$. If a separate decoy “quality” parameter $q_{k}$ is retained, it is interpreted as a design parameter that shifts either $\pi_{k}$ (the likelihood of engaging type k) and/or $e_{k}$ (the likelihood of detection conditional on engagement), but it is not multiplied as an additional independent probability unless explicitly modeled; in the linearity proof and the resulting LP, the detection contribution depends on $\pi_{k}e_{k}$ as the effective event probability.

Finally, $B_{D}$ denotes the defender’s per-detection benefit (e.g., intelligence gain, containment benefit, or attribution value), consistent with the defender-utility definition in Section 3.2.1. For clarity, $B_{D}$ is treated as a constant in Section 5 because the theorem concerns allocation structure under linear detection benefit; heterogeneous $B_{D,k}$ can be incorporated without changing the proof form by replacing $B_{D}$ with type-dependent coefficients.

### 5.1. The Defender’s Optimization Problem

The defender’s problem is to choose an allocation, n, that maximizes expected utility, subject to a budget constraint B, as follows:

(24) $$\underset{n}{\max E}\;\left[U_{D}\left(n\right)\right]$$

$\mathrm{Subject}\;\mathrm{to}\;\sum_{k=1}^{K}c_{k}n_{k}\leq B\;and\;n_{k}\geq 0\;\mathrm{for}\;\mathrm{all}\;k.$

### 5.2. Theorem 5: Greedy Allocation Property

The theorem characterizes a structural property of the optimal allocation. Under fixed attacker interaction probabilities that do not depend on the number of deployed decoys, an optimal allocation exists that concentrates investment in a single decoy type with the highest “bang-for-the-buck”.

**Theorem** **5.** *In the heterogeneous deception game (HDG), if the attacker chooses among decoy types with a fixed probability $q_{k}$ for each type k, independent of the number of decoys $n_{k}$, and if $n_{k}$ are continuous decision variables with $n_{k}\geq 0$, then there exists an optimal allocation strategy where the defender only invests in a decoy type with the highest ratio $(q_{k}e_{k})/c_{k}$. In particular, for any of the following:*

(25) $$k^{*}\in arg\;\underset{k}{max}\frac{q_{k}e_{k}}{c_{k}},$$

One optimal allocation is as follows:

(26) $$n_{k^{*}}=\frac{B}{c_{k^{*}}},n_{k}=0\;\mathrm{for}\;\mathrm{all}\;k\neq k^{*}$$

#### 5.2.1. Formal Proof

**Lemma** **1.** *The defender’s expected utility function $E[U_{D}(n)]$ is a linear function of the number of decoys of each type, $n_{k}$.*

**Proof of Lemma** **1.** Under the assumption that the attacker’s probability of interacting with each decoy type is fixed at $q_{k}$, the expected utility from deploying $n_{k}$ decoys of type k is $n_{k}\;q_{k}\;e_{k}\;B_{D}$, where $B_{D}$ is the benefit of detecting an attack. Summing up all types, the total expected utility from detection is as follows:

(27) $$\sum_{k=1}^{K}n_{k}\;q_{k}\;e_{k}\;B_{D}$$

□

This is a linear function of the $n_{k}$ values.

**Proof of Theorem** **5.**

- By Lemma 1, the defender’s optimization problem is the linear program (LP):
  (28) $$\underset{n}{max}\;\sum_{k=1}^{K}n_{k}\;\left(q_{k}e_{k}B_{D}\right)$$
  $\mathrm{Subject}\;\mathrm{to}\;\sum_{k=1}^{K}c_{k}n_{k}\leq B,n_{k}\geq 0.$
- Define the “bang-for-the-buck” for decoy type k as follows:
  (29) $$r_{k}=\frac{q_{k}e_{k}B_{D}}{c_{k}}$$
- For any feasible allocation n, we find the following:
  (30) $$\sum_{k=1}^{K}n_{k}(q_{k}e_{k}B_{D})=\sum_{k=1}^{K}r_{k}\;c_{k}n_{k}\leq \left(\underset{j}{max\;}r_{j}\right)\sum_{k=1}^{K}c_{k}n_{k}\leq \left(\underset{j}{max\;}r_{j}\right)B$$
- Let $k^{*}\in arg\;{max}_{j}r_{j}$. Consider the allocation $n_{k^{*}}=B/c_{k^{*}}$ and $n_{k}=0$ for all $k\neq k^{*}$. This allocation is feasible and achieves objective value, as follows:
  (31) $$r_{k^{*}}B=\left(\underset{j}{max\;}r_{j}\right)B$$
- As the allocation achieves the upper bound in Step 3, it is optimal. Therefore, there exists an optimal allocation that invests only in a decoy type $k^{*}$ with the highest ratio $(q_{k}e_{k})/c_{k}$. □

This result has the following important practical implications:

1. Simplified decision-making: Defenders do not need to solve a complex multi-dimensional optimization problem. They can rank decoy types by $(q_{k}e_{k})/c_{k}$ and invest in a highest-ranked type.
2. Focus on quality: The theorem suggests that it is often better to invest in a smaller number of high-quality, highly effective decoys than to spread resources across many low-quality ones.
3. Sensitivity to attacker behavior: The optimal allocation depends on the attacker’s interaction probabilities $q_{k}$. If these probabilities are not fixed but depend on the defender’s allocation, the problem becomes more complex.

This theorem establishes a tractable structural property of heterogeneous decoy allocation by showing that, under fixed interaction weights, the HDG reduces to a single-index “bang-for-the-buck” rule with an optimal corner allocation in the continuous relaxation. In the context of defender decoy portfolio design, the result provides an interpretable benchmark that clarifies when heterogeneity collapses to a provably optimal single-type investment and when richer attacker–defender coupling is required.

##### Validation of Theorem 5

**Figure 10:**
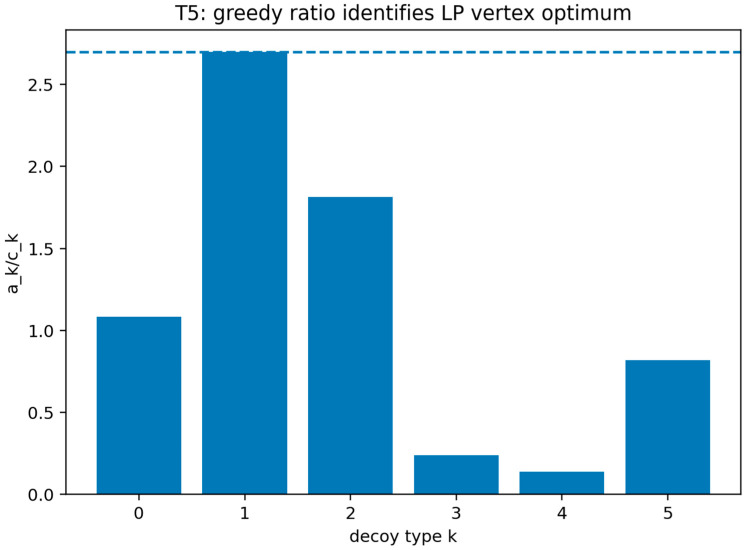
Vertex optimality and greedy allocation verification in the heterogeneous decoy LP regime.

Theorem 5 was validated (see Figure 10) by instantiating the heterogeneous decoy allocation model under the linearity condition that the attacker interaction probability with each decoy type is fixed and independent of deployed quantities. Under this condition, the defender objective is linear and the feasible set is a budget polytope. The validation solves the resulting linear program and checks that an optimal solution is attained at an extreme point that concentrates the budget on the decoy type with the best effectiveness-to-cost contribution to the objective, as characterized by the theorem.

This figure compares the linear-program optimum in the heterogeneous allocation setting to the extreme-point allocation that places the full budget on the best effectiveness-to-cost decoy type. It shows coincidence of the greedy extreme-point solution with the linear-program optimum under the theorem’s linearity assumptions. Vertex optimality and greedy allocation verification in the heterogeneous decoy LP regime. The y-axis reports the defender’s objective value in the continuous relaxation linear program (expected detection benefit minus deployment cost) evaluated for each compared allocation. The figure contrasts (i) the LP-optimal allocation and (ii) the greedy extreme-point (vertex) allocation that assigns the full budget to the decoy type maximizing $e_{k}B_{D}/c_{k}$. The coincidence of the two objective values provides a computational certificate that the greedy vertex attains the LP optimum under the theorem’s linearity assumptions.

## 6. Computational Complexity Analysis

This section analyzes the computational complexity of finding optimal strategies in the proposed game-theoretic framework and delineates the tractability boundary between the basic SDG and heterogeneous allocation variants.

### 6.1. Complexity of the Basic SDG

The basic SDG can be solved by enumerating the defender’s discrete decoy-count decision and evaluating the induced attacker best responses across attacker types. Let the defender’s strategy be the number of deployed decoys, denoted by nS∈{0,1,…,Nmax−NR}, where Nmax is the maximum number of possible targets and NR is the number of real assets. For each candidate nS, the defender computes the expected utility by iterating over attacker types θ∈Θ and evaluating the corresponding best response $a^{*}(nS,\theta )$, followed by aggregation under the prior p(θ).

A generic evaluation procedure is as follows.

- Enumerate defender strategies: for each nS∈{0,1,…,Nmax−NR}.
- Compute best responses: for each θ∈Θ, compute $a^{*}(nS,\theta )$.
- Aggregate expected utility: compute $E[UD(nS)]=\sum_{\theta \in \Theta }p(\theta )\;UD\;\left(nS,a^{*}(nS,\theta )\right)$.

To state complexity precisely, define TBR as the time required to compute one attacker best response and the associated utility contribution for a fixed pair (nS,θ). Then the total runtime of the enumeration procedure is as follows:

(32) O ((Nmax−NR+1) ∣Θ∣ TBR)

O describes the asymptotic order of growth of an algorithm’s running time (or memory) as the problem size increases. This provides an upper bound to constant factors and ignores lower-order terms and means that the runtime grows proportionally to the product of the following:

- $\left(N_{max}-N_{R}+1\right)$: the number of defender strategies being enumerated (each possible decoy count).
- ∣Θ∣: the number of attacker types being evaluated per defender strategy.

#### 6.1.1. Theorem 6: Parameterized Polynomial-Time Solvability of the Basic SDG

**Theorem** **6.** *The basic SDG can be solved in time O ((Nmax−NR+1) ∣Θ∣ TBR). In particular, if the attacker best response for each (nS,θ) is computable in constant time (for example, under a homogeneous target representation in which the attacker’s payoff depends only on whether a chosen target is real versus decoy), then the SDG is solvable in O ((Nmax−NR+1) ∣Θ∣) time [87].*

**Proof.**  The defender evaluates each candidate nS in the finite set {0,1,…,Nmax−NR}, whose cardinality is Nmax−NR+1. For each nS, the defender iterates across the finite set of attacker types Θ. By definition, each (nS,θ) evaluation requires TBR time. Therefore, the total time is the product of these factors, yielding O ((Nmax−NR+1) ∣Θ∣ TBR). Under the stated constant-time best-response condition, TBR=O(1), which implies O ((Nmax−NR+1) ∣Θ∣). This bound is polynomial in $\left(N_{max}-N_{R}+1\right)$ and ∣Θ∣ under the assumption that $T_{BR}$ is constant for the basic SDG representation. □

This theorem provides a transparent tractability statement for the restricted SDG action representation and makes it explicit which modeling choices (e.g., homogeneous versus target-specific payoffs) control whether evaluation is linear or quadratic in the number of targets.

##### Validation of Theorem 6

**Figure 11:**
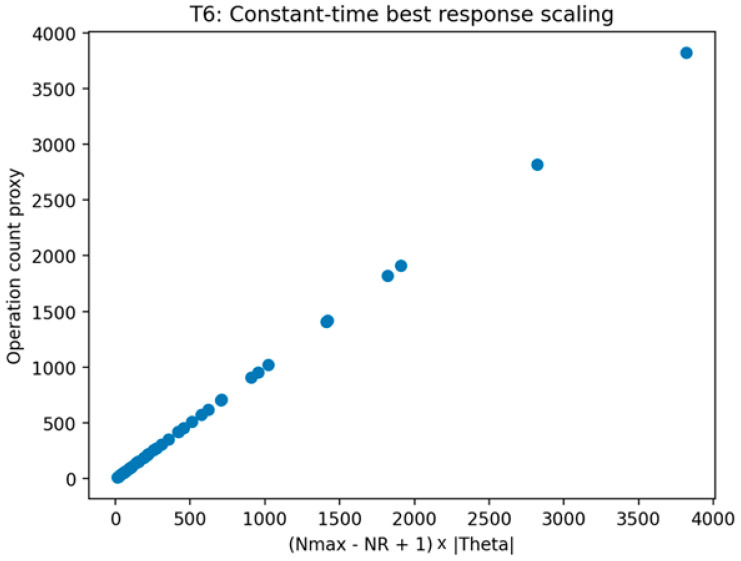
Operation count certificate under constant TBR.

**Figure 12:**
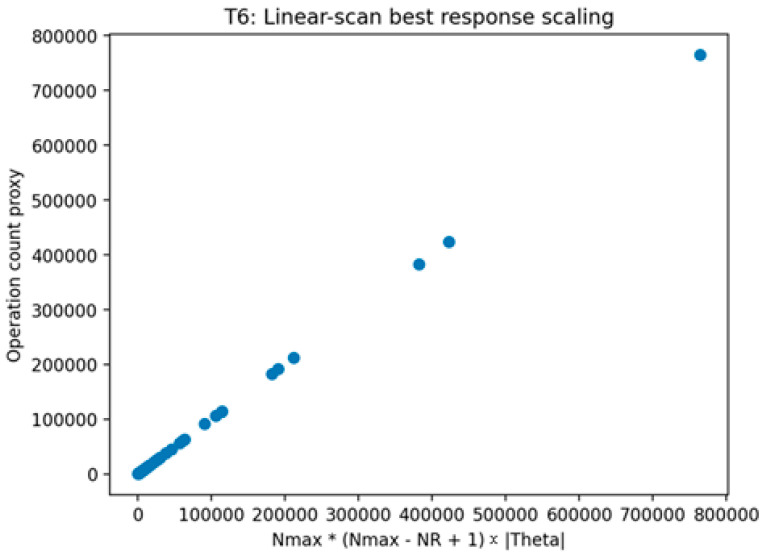
Operation count certificate under linear TBR.

**Figure 13:**
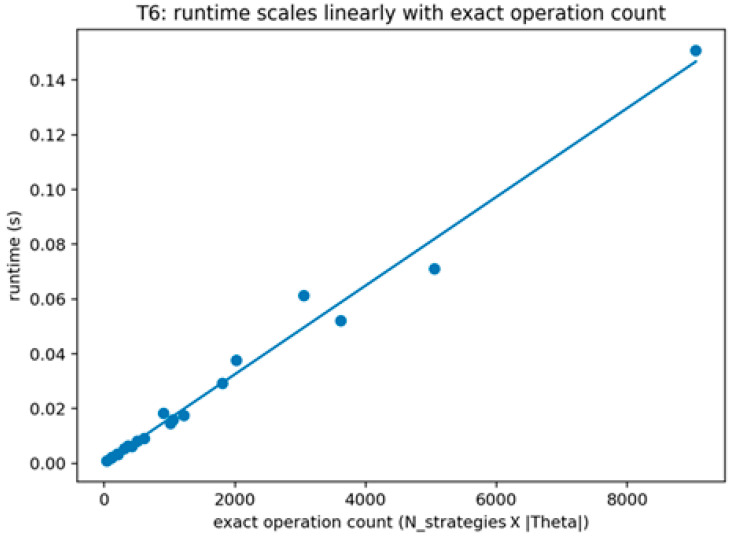
Runtime vs. operation proxy enumeration solver with $N_{max}$ and ∣Θ∣.

Theorem 6 was validated (see Figure 11, Figure 12 and Figure 13) through an operation count certificate that mirrors the implemented enumeration procedure. The validation records the number of feasible defender strategies, multiplies it by the number of attacker types, and parameterizes the per-type best-response evaluation cost by TBR to produce a closed-form evaluation-count expression. The validation is marked as passing when the recorded expression matches the implemented evaluation loops and when empirical scaling plots are consistent with the predicted growth under the tested regimes for TBR.

This figure visualizes the enumeration procedure under a constant-time best-response evaluation regime and shows the resulting scaling as N_max_ and |Θ| vary. The figure provides a concrete illustration consistent with the certificate expression when TBR is treated as constant.

This figure visualizes the enumeration procedure under a linear-scan best-response evaluation regime and shows the resulting scaling as N_max_ and |Θ| vary. The figure provides a concrete scaling illustration consistent with the certificate expression when TBR grows linearly with the number of targets.

This figure plots measured runtime or evaluation steps against the operation count proxy proportional to the product of the number of feasible defender strategies and the number of attacker types. The figure illustrates the implemented enumeration procedure’s scaling behavior consistent with the recorded evaluation count certificate.

### 6.2. Complexity of Extended Models

The computational complexity increases in the extended models because the defender’s decision space and the attacker’s inference/learning mechanisms become higher dimensional, often requiring optimization over information structures, beliefs, or state trajectories.

For signaling-with-evidence extensions, the computational burden typically shifts from enumerating a one-dimensional commitment to optimizing over a signaling or evidence structure and the induced receiver response map, which can be computationally challenging in general formulations. Results in algorithmic persuasion with evidence document that the sender’s optimization task can become difficult and that approximation questions become central in broad input models [88,89].

For dynamic and repeated deception models, the main driver of complexity is the expansion of the state space (configuration states, belief states, or attacker knowledge states). When the defender’s problem is cast as a partially observable or decentralized control process, worst-case complexity can become very high. For example, decentralized control formulations (which capture multi-component systems and partial observability) admit strong intractability results, including very high worst-case complexity classes for finite-horizon variants [38,90].

For bounded rationality via quantal response, computing the equilibrium typically requires solving a nonlinear fixed-point system (or its extensive-form analogs). In extensive-form settings, the agent quantal response equilibrium (AQRE) framework formalizes the fixed-point structure under quantal choice, which motivates the iterative numerical solution methods whose performance depends on the conditioning of the underlying game and the smoothing parameter [91,92].

### 6.3. Scalable Solution Approaches

For large-scale instances, scalable approaches are framed around three complementary strategies. First, approximation algorithms are appropriate when exact optimization is computationally prohibitive, with guarantees stated as bounds on solution quality where such bounds are available. Second, domain-guided heuristics are appropriate when provable approximations are unavailable or too conservative, particularly when defender objectives prioritize interpretability and operational constraints. Third, decomposition is appropriate when the game admits separable structure (e.g., asset-wise decomposition, type-wise decomposition, or weak coupling across subgames), allowing parallel solutions of subproblems followed by coordination at a higher level.

### 6.4. NP-Hardness of Heterogeneous Decoy Allocation Problem (HDAP)

The heterogeneous allocation setting motivates a formal hardness result that explains why the study uses tractable relaxations and greedy-style benchmarks rather than claiming general polynomial-time exactness for integer allocation.

**Definition** **1.** *(Heterogeneous Decoy Allocation Problem (HDAP)). Let there be m decoy types indexed by i∈{1,…,m}. Each type i has a nonnegative cost $c_{i}$ and a nonnegative utility contribution $v_{i}$ (interpreted as the defender’s per-decoy marginal expected benefit in the reduced allocation view). Given a total budget B≥0 and a target utility threshold V≥0, HDAP asks whether there exists an integer allocation vector $n=(n_{1},\ldots ,n_{m})$ with $n_{i}\in N_{0}$ such that (i) the budget constraint holds, $\sum_{i=1}^{m}c_{i}n_{i}\leq B$, and (ii) the utility target is achieved, $\sum_{i=1}^{m}v_{i}n_{i}\geq V$. This decision formulation is the integer allocation counterpart of the continuous relaxation analyzed in Section 5 and is used to formalize the computational boundary of heterogeneous decoy portfolio design.*

(33) $$\sum_{i=1}^{m}c_{i}n_{i}\leq B;\;\mathrm{and}\;\sum_{i=1}^{m}v_{i}n_{i}\geq V$$

**Theorem** **7.** *NP-Hardness of HDAP.*

**Proof.**  A polynomial time reduction is constructed from the unbounded knapsack decision problem. Consider an arbitrary instance of unbounded knapsack with item set {1,…,I}, weights {w1,…,wI}, values {p1,…,pI}, capacity W, and target value P. Construct an instance of HDAP with K=I decoy types by setting ci=wi, vi=pi, B=W, and U=P. Because both problems allow nonnegative integer multiplicities of each type/item, any feasible unbounded knapsack solution with a total weight of at most W and a total value of at least P corresponds directly to an HDAP allocation meeting the budget and utility constraints. Conversely, any HDAP allocation corresponds to a feasible unbounded knapsack selection with the same totals. The construction is clearly polynomial in the size of the knapsack instance, and correctness follows from the one-to-one preservation of feasibility under the mapping. As unbounded knapsack is NP-hard, HDAP is NP-hard [93]. □

This theorem establishes a clean hardness baseline for heterogeneous integer allocation, thereby justifying the emphasis on (i) the continuous relaxation and its single-index structure in Section 5, and (ii) scalable heuristics and approximation-oriented methods when integer constraints and heterogeneity are retained. This hardness result explains why the continuous HDG relaxation admits a simple ratio-based structural solution, while the integer HDAP formulation requires heuristics or approximation methods for large-scale deployment.

Although the NP-hardness follows from a standard knapsack lineage, the reduction is included to formalize the computational boundary of heterogeneous decoy allocation under the above HDAP definition and to justify the focus on scalable relaxations and heuristic policies for large decoy catalogs.

#### Validation of Theorem 7

**Figure 14:**
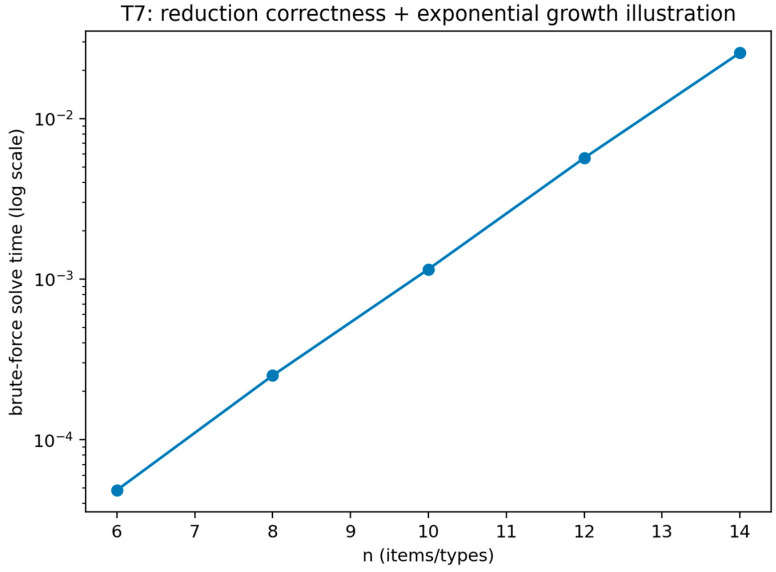
Reduction correctness pass rate across instance sizes.

**Figure 15:**
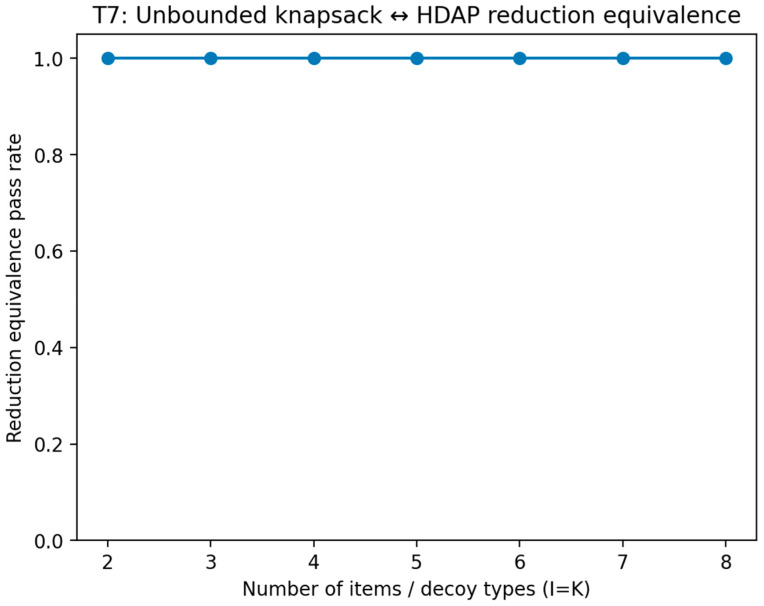
Knapsack-to-HDAP reduction validation and growth diagnostics.

**Figure 16:**
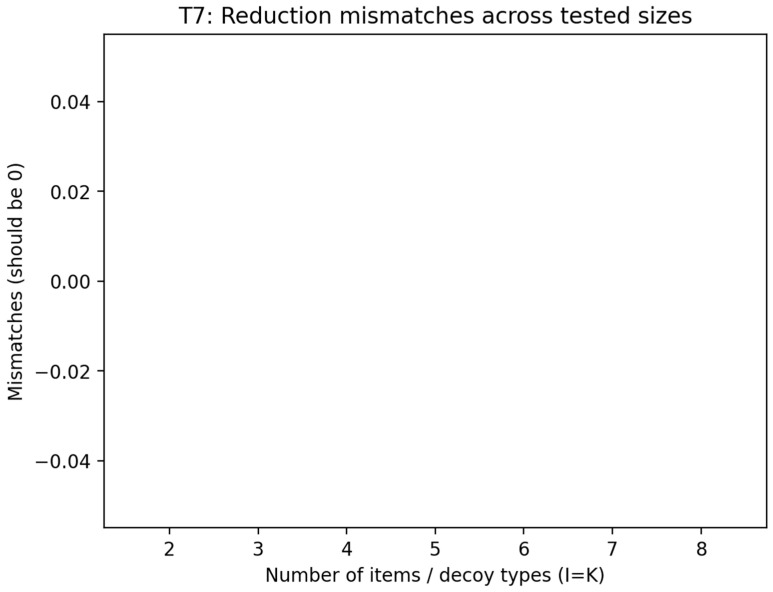
Reduction mismatch count across trials.

Theorem 7 was validated (see Figure 14, Figure 15 and Figure 16) by constructing the reduction mapping used in the hardness argument and checking decision equivalence on a suite of generated instances. The validation maps knapsack weights to decoy-type costs, knapsack values to decoy-type utilities, capacity to the deception budget, and the knapsack target to the HDAP target, then evaluates the feasibility of each paired instance. The validation is marked as passing when feasibility of the knapsack instance agrees with feasibility of the mapped HDAP instance for all tested cases, and when the mapping preserves instance size growth consistent with polynomial time construction.

This figure summarizes reduction tests and instance size growth diagnostics for the mapped decision problems. It illustrates that the reduction mapping behaves consistently across increasing instance sizes in the tested set and by reporting any observed inconsistencies in the mapping-based decision equivalence check.

This figure reports the reduction-correctness pass rate across the tested instance set, typically stratified by instance size. It demonstrates that the knapsack-to-HDAP mapping preserves the decision outcome across the evaluated instances.

This figure reports the number of decision mismatches, if any, between the original knapsack instances and the mapped HDAP instances across the tested set. It supports Theorem 7 by certifying that mismatches are absent in the validated regime, which is the computational counterpart of the reduction’s forward and backward direction checks.

## 7. Information-Theoretic Analysis of Deception

A significant gap in the current literature is the absence of a formal method to quantify the fundamental limits of cyber deception. In this section, we introduce an extension to our framework by defining the concept of deception capacity, an information-theoretic measure that characterizes the maximum amount of uncertainty a defender can induce in an attacker.

### 7.1. Information-Theoretic Foundation

**Figure 17:**
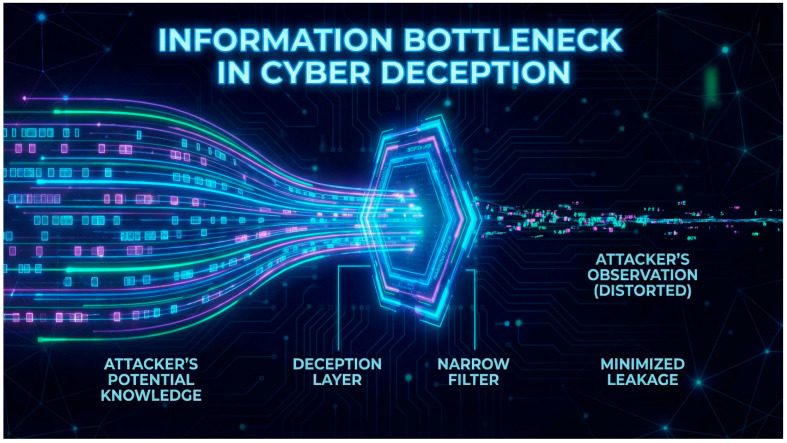
Information leakage.

At its core, cyber deception is a process of manipulating an attacker’s beliefs by controlling the information they receive (see Figure 17). We can model this process using concepts from information theory.

Let T be a random variable representing the target type (real or decoy), and O be the attacker’s observation. The defender’s goal is to maximize the attacker’s uncertainty about T after observing O. This is measured by the conditional entropy H(T∣O). Conversely, the information leakage I(T;O)=H(T)−H(T∣O) represents what the attacker learns, which the defender seeks to minimize.

(34) I(T;O) = H(T) − H(T∣O)

where H(T) is the prior entropy (attacker’s uncertainty before any observation), and H(T∣O) is the conditional entropy (attacker’s remaining uncertainty after observation). A successful deception strategy keeps H(T∣O) close to H(T), meaning the attacker learns little from their observations. Effective deception aims to minimize the information leakage, i.e., minimize I(T;O). The symbol T is used in this section as a random variable representing the target type (real vs. decoy); later references to a set of observed targets should be interpreted separately to avoid overloading the notation.

This foundation frames deception effectiveness as an explicit information-leakage minimization objective, enabling direct comparison across deception mechanisms through a common entropy-based criterion.

### 7.2. Defining Deception Capacity

We define deception capacity as the supremum of the normalized conditional entropy achievable by the defender, subject to constraints on the cost and fidelity of the deception.

Let the set of real assets be R and the set of decoy assets be D. The attacker observes a set of targets T=R∪D. The attacker’s goal is to distinguish between R and D.

We can model the deception system as a communication channel, where the defender’s strategy (the configuration of R and D) is the input, and the attacker’s observation is the output. The channel is characterized by the conditional probability p(o∣s), which represents the probability of the attacker making an observation o, given the defender’s strategy s.

**Definition** **2.** *We define deception capacity as the supremum of the normalized conditional entropy of the target type T (real vs. decoy) given the attacker’s observation O, taken over all admissible defender strategies s∈S, as follows:*

(35) $$C_{D}\;=\;\underset{s\in S}{sup}\left[\frac{H(T|O;s)}{H(T)}\right]$$

*where H(T∣O;s) denotes the conditional entropy of T given O when the defender plays strategy s, and H(T) is the prior entropy of the target type distribution. This ratio is bounded in [0, 1], where $C_{D}=1$ indicates perfect deception (the attacker learns nothing from observation) and $C_{D}=0$ indicates complete information leakage (the attacker can perfectly distinguish real targets from decoys). The defender’s objective is to select the strategy $s^{*}$ that achieves this supremum, thereby maximizing the attacker’s residual uncertainty. This is analogous to the definition of channel capacity in information theory.*

This definition introduces a normalized, capacity-style upper bound that is expressed directly in terms of conditional entropy of real-versus-decoy discrimination, thereby formalizing “fundamental limits” as an optimizable uncertainty ratio over admissible deception strategies.

### 7.3. Implications for Deception Design

The concept of deception capacity provides a powerful tool for the analysis and design of deception systems:

- Benchmarking: Deception capacity provides a theoretical upper bound on the effectiveness of any deception strategy. It can be used to benchmark the performance of practical deception systems.
- Resource allocation: By understanding the factors that influence deception capacity (e.g., the number of decoys, their quality, the attacker’s observational capabilities), more informed decisions about resource allocation can be made.

These implications operationalize the capacity concept as a defender-facing benchmark and allocation lens, connecting information leakage directly to design parameters such as decoy quantity, decoy quality, and attacker observation strength.

### 7.4. Future Research Directions

This extension opens several new avenues for research:

- Calculating deception capacity: Developing algorithms to calculate or approximate the deception capacity for different types of deception systems.
- Achieving deception capacity: Designing practical deception strategies that can achieve the theoretical deception capacity.
- Dynamic deception capacity: Extending the concept to dynamic and adaptive deception scenarios.

By providing a formal, information-theoretic foundation for cyber deception, this extension paves the way for a more rigorous and systematic approach to the design and analysis of deception systems. These directions extend the framework from static characterization to computability, achievability, and dynamic generalization, positioning deception capacity as a unifying objective for both theoretical limits and deployable strategy design.

#### 7.4.1. Calculating Deception Capacity

A directly computable formulation treats deception capacity as a constrained optimization over defender-configurable “channel designs.” Let the target type be T∈{R,D} with prior p(t), the attacker observation be O∈O, and the defender strategy be s∈S. The induced observation model is p(o∣t;s). The defender’s objective is to maximize normalized residual uncertainty, as follows:

(36) $$\underset{s\in S}{max}\;\frac{H\left(T|O;s\right)}{H\left(T\right)}⟺\underset{s\in S}{\min I}\left(T;O;s\right)\;$$

because H(T) is fixed and I(T;O;s)=H(T)−H(T∣O;s).

One canonical choice for replacing the abstract strategy space S with explicit decision variables that parameterize feasible observation channels is to optimize over a confusion matrix $Q_{s}$ with entries $Q_{s}(o|t)$ that must satisfy (i) probability simplex constraints $\sum_{o}Q_{s}(o|t)=1$, $Q_{s}(o|t)\geq 0$, and (ii) implementation constraints that encode “cost and fidelity,” for example, as follows:

(37) $$\sum_{t}\sum_{o}p\left(t\right)\;Q_{s}\left(o|t\right)\;c\left(t,o\right)\;\leq B$$

(38) $$\sum_{o}Q_{s}\left(o|t\right)\;l\left(t,o\right)\;\leq \;\varepsilon_{t}\;\forall t$$

where c(t,o) is a cost model and l(t,o) encodes fidelity/leakage limits that the defender cannot violate (e.g., constraints on how much the distribution of decoy observations can deviate from real observations). With $Q_{s}$ as the decision variable, the objective I(T;O) becomes a smooth function of $Q_{s}$ via the following:

(39) $$p(o)=\sum_{t}p(t)\;Q_{s}(o|t),I(T;O)={\sum_{t}\sum_{o}p(t)\;Q}_{s}(o|t)log\frac{Q_{s}(o|t)}{p(o)}$$

This yields a standard constrained information optimization problem. When the feasible set over $Q_{s}$ is convex (common for linear cost/fidelity constraints), the resulting program is amenable to Lagrangian methods and iterative scaling schemes. In discrete settings, a practical algorithmic path is to adapt the classical channel capacity computation machinery (iterative maximization/minimization of mutual information under constraints), which is historically exemplified by iterative algorithms for channel capacity and constrained variants. A useful modern technical bridge for cost-constrained mutual information optimization is the explicit treatment of maximizing (generalized) mutual information under cost constraints and exponential average constraints, which provides a defensible template for dual-based computation [94].

This converts “capacity” into a concrete constrained optimization with an implementable feasible region, enabling (a) exact solutions for small alphabets, (b) convex relaxations when the channel set is convex, and (c) sample average approximations when p(o∣t;s) is accessible only through simulation.

#### 7.4.2. Achieving Deception Capacity

Instead of merely computing the supremum, this designs a family of strategies guaranteed to approach the computed upper bound under explicit engineering constraints. This is most cleanly posed as a joint design of (a) decoy configuration variables and (b) a measurable “indistinguishability mechanism” that shapes the attacker’s observation channel.

A tractable formulation introduces a parametric strategy s(x) governed by design variables x∈X (e.g., feature templates, traffic-shaping parameters, surface signatures, response timing distributions). The induced channel is p(o∣t;x). Achievability is then posed as the constrained minimization, as follows:

(40) $$\underset{x\in X}{\min I}\;\left(T;O;x\right)\;s.t.\;g\left(x\right)\leq 0,\;h\left(x\right)\leq B$$

With g(x) capturing fidelity/operational constraints and h(x) capturing cost.

If the defender does not know the attacker’s true observation model but knows it lies in an ambiguity set P(x), then the defender can design for worst-case leakage, as follows:

(41) $${min}_{{x\in X}^{{max}_{p\in P\left(x\right)}}}I_{p}\left(T;O;x\right)$$

The designed $x^{⋆}$ guarantees a bounded leakage under all channels in $P(x^{⋆})$, and therefore guarantees a lower bound on $H(T|O;x^{⋆})$, [95]. The practical mechanism to “achieve capacity” is thus to compute a capacity benchmark from Section 7.4.1; solve the robust or nominal design problem above; and report an achievability gap, as follows:

(42) $$\Delta \left(x\right)=\frac{H\left(T|O;x\right)}{H\left(T\right)}-C_{D}$$

or its leakage equivalent $I(T;O;x)-I^{⋆}$. Thus, this is not about the existence of a mutual-information objective, but the explicit end-to-end pipeline that (1) defines a benchmark, (2) designs a feasible strategy family, and (3) reports a certifiable gap under constraints.

#### 7.4.3. Dynamic Deception Capacity

Let $T_{\tau }$ be the target type (or configuration-relevant hidden variable) at time τ, and $O_{0:\tau }$ be the history of attacker observations. A natural dynamic analogue is to consider the attacker’s residual uncertainty after τ steps, $H(T_{\tau }|O_{0:\tau })$, or the cumulative information leakage $\sum_{\tau }I(T_{\tau };O_{\tau }|O_{0:\tau -1})$. A dynamic deception capacity can be defined over defender policies π that map histories (or beliefs) into actions, as follows:

(43) $$C_{D}^{\left(\tau \right)}={sup}_{\pi }\frac{H(T_{\tau }|O_{0:\tau };\pi )}{H(T_{0})}$$

Subject to per-period or cumulative cost constraints.

This becomes a partially observable Markov decision process (POMDP) problem in which the defender’s state includes (i) the system configuration and (ii) a belief about the attacker’s knowledge. The reward is information-theoretic: rather than maximizing mutual information (as in active information gathering), the defender minimizes leakage or maximizes attacker uncertainty. The methodological template is well established in POMDP planning with mutual-information criteria and sample-based approximations, which shows how information quantities can be embedded as objective functions within sequential decision-making [96]. A directly implementable method is a receding-horizon policy, as follows:

- At time τ, compute the defender belief state $b_{\tau }$ over relevant hidden variables.
- Choose an action $u_{\tau }$ that minimizes predicted leakage over a horizon H, as follows:
  (44) $$\underset{u_{\tau :\tau +H-1}}{\min E}\;[\sum_{k=0}^{H-1}I(T_{\tau +k};O_{\tau +k}|O_{0:\tau +k-1})]+\lambda \;\mathrm{Cos}t(u_{\tau :\tau +H-1})$$
- Execute $u_{\tau }$, observe feedback, update the belief, and repeat.

**Figure 18:**
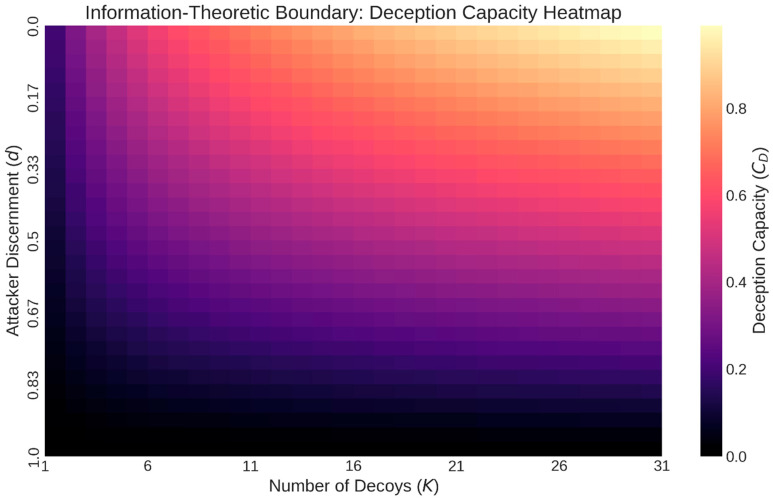
Deception capacity heatmap.

This yields “dynamic deception capacity” as a policy-dependent performance (see Figure 18) limit and provides a principled bridge to adaptive deception (decoy rotation, reconfiguration, staged exposure). As a conceptual reinforcement for treating information terms as resources in sequential control, recent work on information-theoretic perspectives in control under partial observability provides a defensible foundation for incorporating information penalties/constraints into sequential optimization objectives [97].

This extension introduces a formal, channel-style limit concept for cyber deception, one that is stated directly in terms of attacker belief uncertainty and information leakage, thereby enabling upper-bound benchmarking independent of any specific decoy implementation. By defining deception capacity as a normalized conditional-entropy supremum over admissible defender strategies, this section establishes an information-theoretic upper bound for cyber deception that enables benchmarking independent of specific decoy implementations. It further positions deception capacity as an optimization object by outlining (i) a constrained program for computing or approximating capacity under cost and fidelity limits, (ii) an implementable design formulation that targets achievability through leakage minimization with explicit performance gaps, and (iii) a dynamic extension that treats deception capacity as a policy-dependent quantity in adaptive and sequential deception settings.

## 8. The VoD Framework

To formally analyze the fundamental value of deception, a game-theoretic framework is introduced that explicitly compares a game with deception to an equivalent game without it [7,98]. This allows the precise benefit that deception provides to be quantified through a paired-game interface (TSG vs. DSG) designed for ROI comparability across different deception scenarios.

### 8.1. The Baseline: TSG

A standard Stackelberg security game [74,99], the TSG, is defined as a baseline where no deception is used.

- Players: A defender (leader) and an attacker (follower).
- Targets: A set of N real targets, $T=\{t_{1},\ldots ,t_{N}\}$.
- Defender’s strategy: The defender has M security resources to allocate. A pure strategy is an allocation of these resources to a subset of targets. The defender commits to a mixed strategy x, which is a probability distribution over all possible pure strategies.
- Attacker’s strategy: The attacker observes the defender’s mixed strategy x and chooses a single target $t_{i}$ to attack.
- Payoffs:
  - If the attacker attacks target $t_{i}$ and it is covered by a resource, the defender receives a reward $R_{D}^{c}$ and the attacker receives a penalty $P_{A}^{c}$
  - If the attacker attacks target $t_{i}$ and it is not covered, the defender receives a penalty $P_{D}^{u}$ and the attacker receives a reward $R_{A}^{u}$
- Equilibrium: The solution concept is the SSE, where the defender chooses the mixed strategy x that maximizes the defender’s expected utility, assuming the attacker will break ties in the defender’s favor.

Let $U_{D}^{*}(TSG)$ be the defender’s optimal expected utility in the SSE of the TSG.

### 8.2. DSG: TSG Extension

The DSG extends the TSG by allowing the defender to deploy decoys [100,101].

- Players and targets: Same as the TSG, but the defender can also deploy K decoys, $D=\{d_{1},\ldots ,d_{K}\}$. The attacker sees a set of N+K potential targets.
- Defender’s strategy: The defender’s strategy involves both allocating M resources to the N real targets and deploying K decoys. The decoys have a deployment cost $c_{d}$ each.
- Attacker’s strategy: The attacker observes the mixed strategy over the real targets and the presence of the decoys, but cannot distinguish real targets from decoys with certainty. The attacker chooses one of the N+K potential targets to attack.
- Payoffs:
  - Payoffs for attacking real targets are the same as in the TSG.
  - If the attacker attacks a decoy $d_{j}$, the defender receives a high reward $R_{D}^{decoy}$ (for detecting the attacker) and the attacker receives a high penalty $P_{A}^{decoy}$.
- Equilibrium: The solution concept is again the SSE.

Let $U_{D}^{*}(DSG)$ be the defender’s optimal expected utility in the SSE of the DSG.

### 8.3. Formulating the VoD

This framework allows the VoD to be formally defined as the ratio of the defender’s optimal utility in the DSG to the defender’s optimal utility in the TSG, as follows:

(45) $$VoD=\frac{U_{D}^{*}(DSG)}{U_{D}^{*}(TSG)}$$

This metric captures the multiplicative improvement in the defender’s utility gained by using deception. A VoD>1 indicates that deception is beneficial, while a VoD=1 indicates that deception provides no value in that specific game. The price of transparency (PoT) is also defined as the absolute difference in utility.

(46) $$PoT=U_{D}^{*}(DSG)-U_{D}^{*}(TSG)$$

Validity condition: The ratio definition is used under $U_{D}^{*}(TSG)>0$. VoD is a dimensionless ratio reporting the multiplicative improvement in defender equilibrium utility obtained when moving from the transparent baseline (TSG) to the deception-enabled game (DSG). As a ratio, VoD is most appropriate when cross-scenario comparability is required across settings with different absolute payoff scales and when the baseline defender equilibrium utility is strictly positive, ensuring the ratio is well-defined and not distorted by sign effects. By contrast, PoT is an absolute difference in utility (measured in the same units as the payoffs) that directly quantifies the marginal utility lost as transparency or observability increases. PoT is therefore most appropriate when the magnitude of the loss matters operationally (e.g., budgeting, cost accounting, and absolute ROI), or when baseline utilities may be near zero, in which case ratios can become numerically unstable or difficult to interpret. Retaining both measures is deliberate: VoD supports normalized cross-setting comparisons, whereas PoT supports additive accounting and sensitivity statements in payoff units; taken together, they reduce the risk of misleading conclusions that can arise when only a single normalization is reported.

#### 8.3.1. VoC Curve and Marginal VoD

This framework also allows the VoD to be evaluated as a function of the number of decoys, thereby defining a deception value curve.

Let $U_{D}^{*}(DSG,K)$ denote the defender’s optimal expected utility in the SSE of a DSG with K decoys and decoy deployment cost $c_{d}$ each. The SSE interpretation is used: the defender commits first, the attacker best-responds after observing the commitment, and ties are broken in the defender’s favor.

Let $S_{K}$ be the defender’s feasible commitment set in the DSG with K decoys (a mixed strategy over allocations of M security resources to the N real targets together with the presence of K decoys). Let $A_{K}=T\cup D_{K}$ be the attacker action set, where $T=\{t_{1},\ldots ,t_{N}\}$ and $D_{K}=\{d_{1},\ldots ,d_{K}\}$. For any defender commitment $x\in S_{K}$, let $BR(x,K)\subseteq A_{K}$ denote the attacker best-response set under the DSG payoffs, and let $a^{*}(x,K)\in BR(x,K)$ be the attacker action selected under SSE tie-breaking in the defender’s favor. Let $U_{D}(x,a;K)$ denote the defender’s expected utility induced by commitment x, attacker action a, and K decoys.

Under this notation, the SSE value with K decoys is as follows:

(47) $$U_{D}^{*}\left(DSG,K\right)=\underset{x\in S_{K}}{max}\;U_{D}(x,a^{*}\left(x,K\right);K)$$

A cost-consistent total utility is also defined, using the linear decoy deployment cost model, as follows:

(48) $$U_{D}^{total,*}(DSG,K)=U_{D}^{*}(DSG,K)-K\;c_{d}$$

Let $U_{D}^{*}(TSG)$ be the defender’s optimal expected utility in the SSE of the TSG. Therefore VoC curve is given by the following:

(49) $$VoD\left(K\right)=\frac{U_{D}^{total,*}\left(DSG,K\right)}{U_{D}^{*}\left(TSG\right)},\;U_{D}^{*}\left(TSG\right)>0$$

PoT curve is given by the following:

(50) $$PoT(K)=U_{D}^{total,*}(DSG,K)-U_{D}^{*}(TSG)$$

Marginal VoD is given by the following:

(51) $$\Delta_{D}(K)=U_{D}^{total,*}(DSG,K)-U_{D}^{total,*}(DSG,K-1),K\geq 1$$

Marginal PoT is given by the following:

(52) $$\Delta_{T}(K)=PoT(K)-PoT(K-1),K\geq 1$$

These definitions ensure that VoD(K), PoT(K), $\Delta_{D}(K)$, and $\Delta_{T}(K)$ are evaluated using the same equilibrium concept (SSE), the same attacker action set $A_{K}$, and the same linear decoy deployment cost $Kc_{d}$ within the DSG.

#### 8.3.2. Budgeted Deception and ROI-Comparable Deployment Interface

The defender’s strategy in the DSG is evaluated under a budget constraint on deception. Let B be a deception budget and let the decoy deployment cost be $c_{d}$ each. The feasible number of decoys satisfies the following:

(53) $$K\cdot c_{d}\leq B$$

Let $K_{max}=\left\lfloor \frac{B}{c_{d}}\right\rfloor$. The defender’s objective is to select $K\in \{0,1,\ldots ,K_{max}\}$ and the associated commitment that maximizes the defender’s total utility in the SSE of the DSG, as follows:

(54) $$K^{*}\in \arg\underset{K\in \left\{0,1,\ldots ,K_{max}\right\}}{\max U_{D}^{total,*}\left(DSG,K\right),}$$

where

(55) $$U_{D}^{total,*}(DSG,K)=U_{D}^{*}(DSG,K)-K\cdot c_{d}.$$

### 8.4. Positioning of This Framework

This framework adapts and operationalizes the concepts of VoD and PoT for the specific context of synthetic cyber deception games. The contribution is their formal operationalization within a paired-game (TSG vs. DSG) evaluation interface designed for ROI comparability across different deception scenarios. This framework provides a principled way to achieve the following:

- Quantify the benefit of deception across different game settings using a standardized metric.
- Identify the conditions under which deception is most and least effective.
- Derive tight theoretical bounds on the maximum possible value of deception.

This sets the stage for a rigorous theoretical analysis of the fundamental value of deception in cybersecurity.

### 8.5. Theorems on VoD

Building on this framework, original theorems are proved that characterize the fundamental limits of deception. These theorems provide formal bounds on the VoD in security games.

#### 8.5.1. Theorem 8: The High-Cost-of-Deception Theorem

This theorem establishes a simple but important condition under which deception provides no value.

**Theorem** **8.** *If the cost of a single decoy,* $c_{d}$*, is greater than the maximum possible marginal gain in utility from deploying that decoy, then the optimal strategy for the defender in the DSG is to deploy zero decoys, and thus the VoD is at most* 1.

**Proof.** 

- Let $U_{D}(K)$ be the defender’s optimal expected utility in a DSG with K decoys, not including the cost of the decoys.
- The total utility for the defender with K decoys is $U_{D}^{total}(K)=U_{D}(K)-K\cdot c_{d}$.
- The defender will only choose to deploy the first decoy if the utility of doing so is greater than the utility of deploying zero decoys. That is, $U_{D}^{total}(1)>U_{D}^{total}(0)$.
- Substituting the definitions, $U_{D}(1)-c_{d}>U_{D}(0)-0\cdot c_{d}$.
- This simplifies to $c_{d}<U_{D}(1)-U_{D}(0)$. Let $G_{1}=U_{D}(1)-U_{D}(0)$ be the marginal utility gain from the first decoy.
- If $c_{d}>{max}_{K}(U_{D}(K)-U_{D}(K-1))$, then $U_{D}^{total}(K)-U_{D}^{total}(K-1)=(U_{D}(K)-U_{D}(K-1))-c_{d}<0$ for all K≥1, so the optimal number of decoys is 0.
- With 0 decoys, the DSG is equivalent to the TSG, so $U_{D}^{*}(DSG)=U_{D}^{*}(TSG)$, and VoD=1. □

##### Validation of Theorem 8

**Figure 19:**
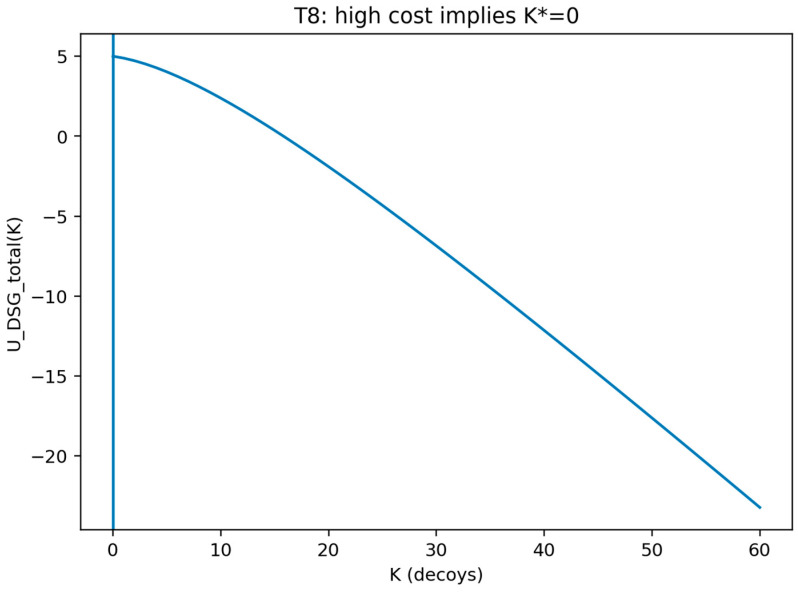
High cost of deception regime: optimality of deploying zero decoys.

**Figure 20:**
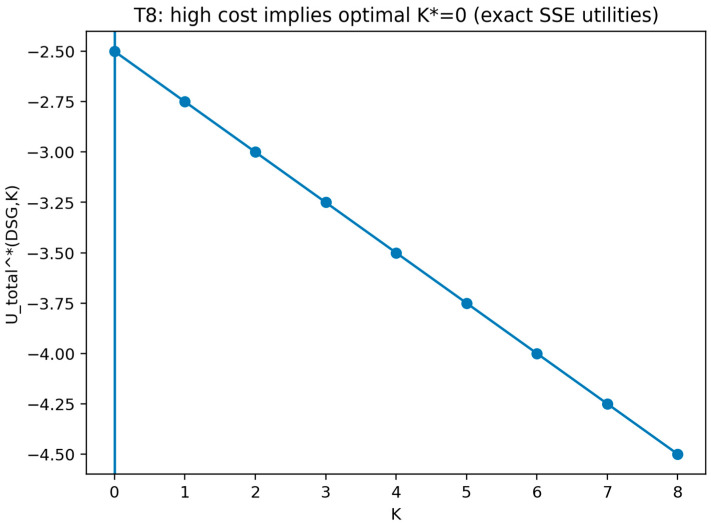
Total utility maximization yields $K^{*}=0$ under prohibitive decoy cost.

Theorem 8 was validated (see Figure 19 and Figure 20) by computing the defender total utility over feasible decoy counts under an explicit per-decoy cost and comparing the marginal utility gain from each additional decoy to that cost. The certificate records the maximum marginal gain, the cost level, and the maximizing decoy count. The validation is marked as passing when the maximizing decoy count is zero in the regime where the per-decoy cost exceeds the maximum marginal gain, which matches the theorem’s prohibitive-cost condition.

This figure plots the defender total utility as a function of the number of deployed decoys under a high per-decoy cost regime. The figure shows that the total utility is maximized at zero decoys when the cost exceeds the maximum marginal benefit from additional decoys.

This figure highlights the optimality of the zero-decoy decision in the high-cost regime, typically by marking the maximizer and the decline in total utility for positive decoy counts. The figure provides an explicit visual witness of K* = 0 under the theorem’s prohibitive-cost condition.

#### 8.5.2. Theorem 9: Budgeted Optimality and Diminishing Returns Condition

**Theorem** **9.** *If* $\Delta_{D}(K)$ *is non-increasing in* K *on the feasible set* $K\in \{1,\ldots ,K_{max}\}$*, then there exists an optimal number of decoys* $K^{*}\in \{0,1,\ldots ,K_{max}\}$ *such that* $K^{*}$ *is the largest feasible* K *satisfying* $\Delta_{D}(K)\geq 0$.

**Proof.** 

- The defender’s total utility with K decoys is $U_{D}^{total,*}(DSG,K)$ with feasibility constraint $K\cdot c_{d}\leq B$, equivalently $K\in \{0,1,\ldots ,K_{max}\}$.
- The increment from K−1 to K is
  (56) $$U_{D}^{total,*}(DSG,K)-U_{D}^{total,*}(DSG,K-1)=\Delta_{D}(K)$$
- If $\Delta_{D}(K)<0$, then $U_{D}^{total,*}(DSG,K)<U_{D}^{total,*}(DSG,K-1)$, so deploying the K-th decoy decreases total utility.
- If $\Delta_{D}(K)$ is non-increasing in K, then for any $K^{\prime }>K$, $\Delta_{D}(K^{\prime })\leq \Delta_{D}(K)<0$, so all subsequent increments also decrease total utility.
- Therefore, among feasible $K\in \{0,1,\ldots ,K_{max}\}$, the optimal number of decoys is attained at the largest feasible $K^{*}$ satisfying $\Delta_{D}(K)\geq 0$. □

##### Validation of Theorem 9

**Figure 21:**
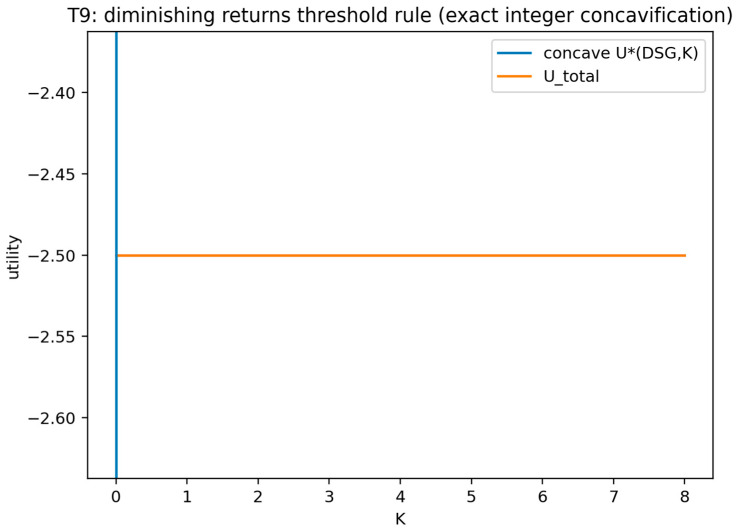
Diminishing returns and the optimal decoy count threshold.

**Figure 22:**
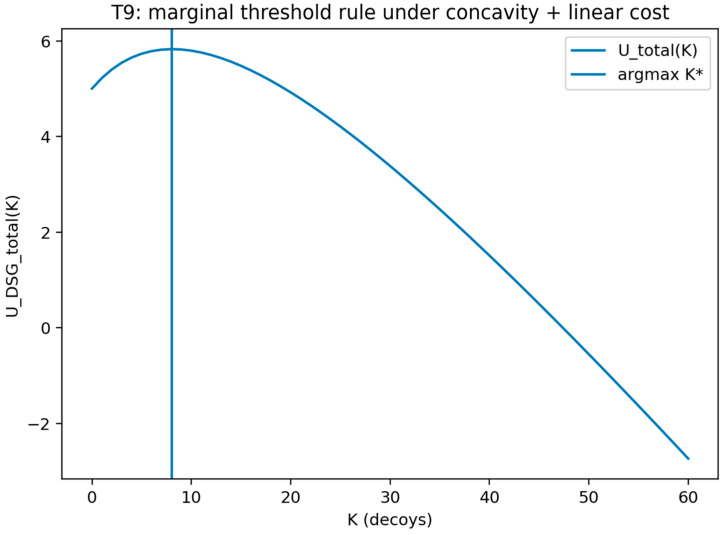
Marginal gain–cost threshold crossing and the optimal $K^{*}$.

Theorem 9 was validated (see Figure 21 and Figure 22) in a diminishing-returns regime in which marginal gains from additional decoys are non-increasing and the defender selects an integer decoy count under a linear per-decoy cost. The validation computes the marginal gains sequence, identifies the first index at which marginal gain falls below cost, and computes the argmax of total utility over the feasible decoy counts. The validation is marked as passing when the argmax coincides with the marginal threshold stopping rule and when the marginal gains sequence satisfies the non-increasing condition used by the theorem.

This figure displays diminishing returns in the benefit of additional decoys, often by plotting the benefit curve or total utility curve and emphasizing concavity or decreasing increments. The figure illustrates the regime in which a marginal threshold characterization is appropriate.

This figure plots the marginal gains from additional decoys together with the per-decoy cost threshold and marks the predicted stopping point. The figure shows agreement between the threshold rule and the computed argmax decoy count in the diminishing-returns regime.

### 8.6. Tight Bounds and Characterization Results

#### 8.6.1. Theorem 10: Upper Bound on the Value of Deception (VoD) Curve

A tight upper bound on the VoD curve is provided under a single attacker type, a single attack, linear decoy deployment cost $c_{d}$, and $U_{D}^{*}(TSG)>0$, as follows:

(57) $$VoD\left(K\right)=\frac{U_{D}^{total,*}\left(DSG,K\right)}{U_{D}^{*}\left(TSG\right)},U_{D}^{total,*}(DSG,K)=U_{D}^{*}(DSG,K)-K\cdot c_{d}$$

**Theorem** **10.** *In any security game with a single attacker type, a single attack, and linear decoy deployment cost $c_{d}$, with $U_{D}^{*}(TSG)>0$, the value of deception satisfies, for all feasible K, the following:*

(58) $$VoD\left(K\right)\leq max(1,\frac{U_{D}^{total,*}\left(DSG,K\right)}{U_{D}^{*}\left(TSG\right)})$$

**Proof.** 

- In the DSG with K decoys, the attacker chooses one of the N+K potential targets to attack, so exactly one outcome is realized: a real target is attacked or a decoy is attacked.
- If a real target is attacked, the defender’s payoff is governed by the same real-target payoffs as in the TSG. Under SSE, the defender’s expected utility from the best achievable real-target outcome is bounded above by $U_{D}^{*}(TSG)$.
- If a decoy is attacked, the defender receives $R_{D}^{decoy}$. Under linear decoy deployment cost, at least one decoy cost $c_{d}$ is incurred whenever a decoy is deployed, so the defender’s payoff from a decoy attack outcome is at most $R_{D}^{decoy}-c_{d}$. If $R_{D}^{decoy}-c_{d}<0$, a decoy attack outcome yields no positive contribution relative to the baseline, so $max\{0,\;R_{D}^{decoy}-c_{d}\}$ is used.
- Therefore, the defender’s optimal total utility in the DSG with K decoys is bounded above by the maximum of the best achievable real-target equilibrium utility and the best achievable decoy-attack payoff, as follows:
  (59) $$U_{D}^{total,*}(DSG,K)\leq max(U_{D}^{*}\left(DSG,K\right),max\{0,\;R_{D}^{decoy}-c_{d}\})$$
- Dividing both sides by $U_{D}^{*}(TSG)>0$ yields the following:
  (60) $$VoD\left(K\right)=\frac{U_{D}^{total,*}\left(DSG,K\right)}{U_{D}^{*}\left(TSG\right)}\leq max(1,\frac{U_{D}^{total,*}\left(DSG,K\right)}{U_{D}^{*}\left(TSG\right)})$$
  □

##### Validation of Theorem 10

**Figure 23:**
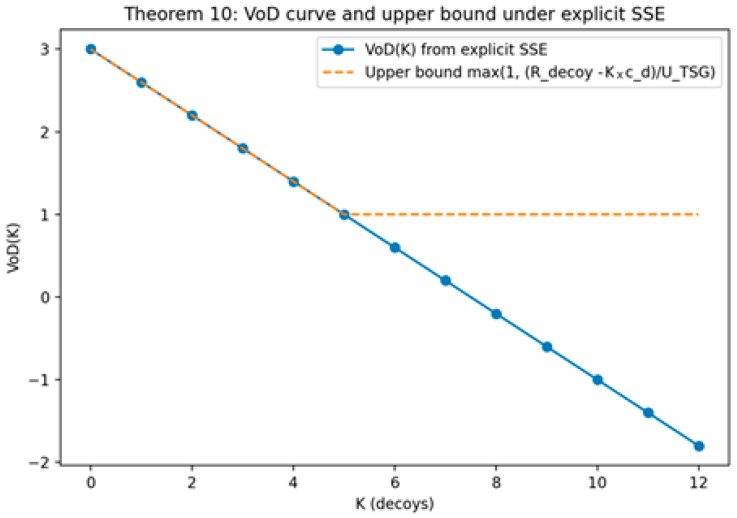
VoD curve and upper bound under explicit SSE.

**Figure 24:**
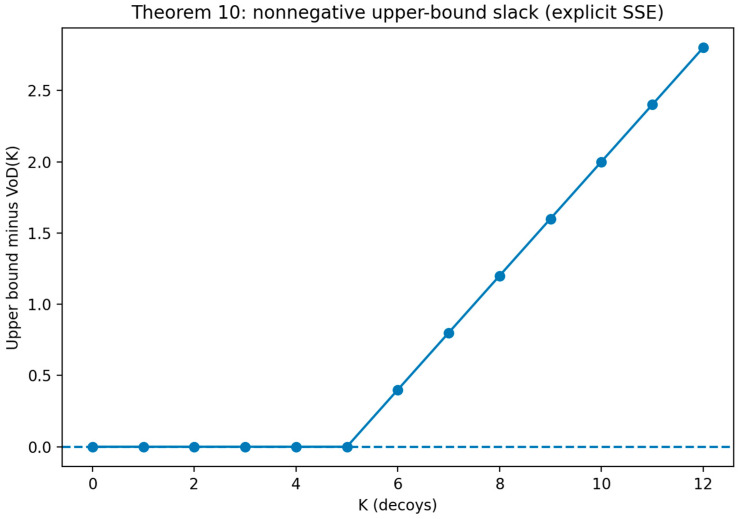
Nonnegative upper-bound gap under explicit SSE.

Theorem 10 was validated (see Figure 23 and Figure 24) by computing exact SSE utilities for the TSG and the corresponding DSG at each decoy count K, under the theorem’s stated modeling conditions (single attacker type; single attack; linear decoy cost $Kc_{d}$; and $U_{D}^{*}(TSG)>0$). The validation then evaluated the $VoD(K)=U_{D}^{*,total}(DSG,K)/U_{D}^{*}(TSG)$ against the theorem’s upper bound pointwise envelope $max\;\left(1,\;(R_{D}^{decoy}-Kc_{d})/U_{D}^{*}(TSG)\right)$ function. The SSE computation was performed by enumerating the attacker’s candidate best response (each real target and the decoy option), solving the defender’s linear program for each candidate response under the attacker best-response inequalities, and selecting the defender-optimal outcome consistent with the SSE tie-breaking convention.

This graph plots VoD(K) computed from explicit SSE utilities for the DSG (including the linear cost $Kc_{d}$) divided by the explicit SSE utility for the TSG. The graph overlays the theorem’s upper bound curve $max\;\left(1,\;(R_{D}^{decoy}-Kc_{d})/U_{D}^{*}(TSG)\right)$ evaluated using the same payoff parameters and the same $U_{D}^{*}(TSG)$ obtained from the SSE solver. The plotted range shows that the computed VoD(K) remains everywhere at or below the bound curve for all tested K, with equality occurring only when the equilibrium outcome achieves the bound under the same feasibility conditions used in the solver run.

This graph plots the pointwise difference (upper bound−VoD(K)) across the same decoy counts K, using the same SSE-computed values as above. The horizontal reference at zero makes the theorem check explicit: all plotted values are nonnegative, which is exactly the inequality condition required by Theorem 10. Where the curve touches zero, the bound is achieved for that K; where it is strictly positive, the bound is conservative for that K.

#### 8.6.2. Theorem 11: Characterization of When Deception Is Ineffective

A formal characterization is provided for the conditions under which deception provides no value (i.e., VoD(K)=1 on the VoD curve defined in Section 8.3.1).

**Theorem** **11.** *The VoD satisfies VoD(K)=1 for all feasible K if any of the following conditions hold: (a) the attacker is “decoy-immune,” meaning the attacker will never attack a decoy regardless of the defender’s strategy or (b) the cost of deception $c_{d}$ is prohibitively high, as defined in Theorem 8.*

**Proof.** 

- Case (a): Decoy-immune attacker. If the attacker is decoy-immune, the attacker’s strategy set is restricted to the set of real targets T. The presence of decoys has no effect on the attacker’s decision-making. As the decoys provide no benefit, the optimal strategy for the defender is to deploy zero decoys. Thus, $U_{D}^{*}(DSG,0)=U_{D}^{*}(TSG)$, and
  (61) $$U_{D}^{total,*}\left(DSG,0\right)=U_{D}^{*}\left(TSG\right),VoD\left(0\right)=\frac{U_{D}^{total,*}\left(DSG,0\right)}{U_{D}^{*}\left(TSG\right)}=1$$

For any K≥1, deploying decoys adds the cost term $K\cdot c_{d}$ without changing attacker behavior, so the optimal strategy remains to deploy zero decoys, and the DSG reduces to the TSG in equilibrium, yielding VoD(K)=1.

- Case (b): Prohibitively high cost. This follows directly from Theorem 8. If the cost $c_{d}$ of a decoy is higher than the marginal utility gain from deploying it, the optimal number of decoys is 0. The DSG reduces to the TSG, so $U_{D}^{total,*}(DSG,0)=U_{D}^{*}(TSG)$, and therefore VoD(K)=1 under the optimal strategy.

□

##### Validation of Theorem 11

**Figure 25:**
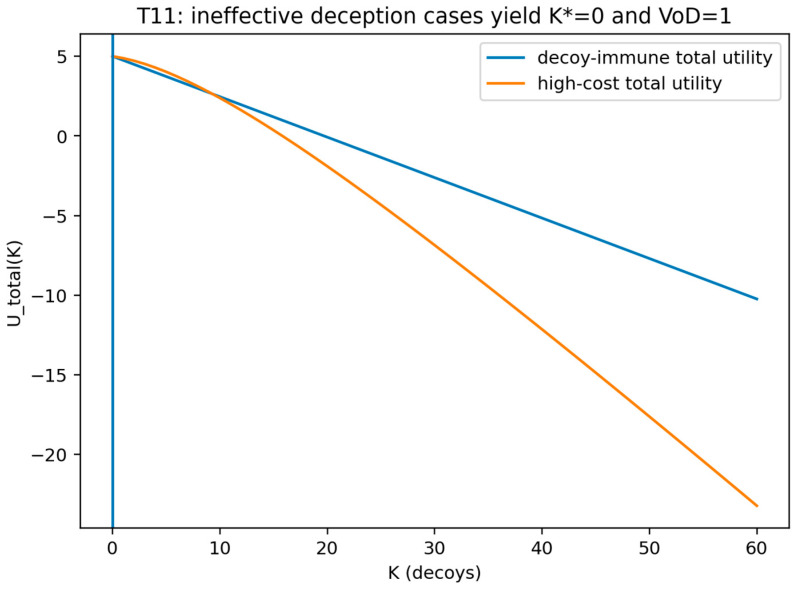
Ineffective deception characterization: decoy-immune and prohibitively high-cost conditions.

Theorem 11 was validated (see Figure 25) by instantiating the ineffective-deception conditions stated in the theorem and solving the defender’s decoy deployment decision under each condition. In the decoy-immune condition, the attacker is restricted to real targets so that decoys cannot change the attacker action; in the prohibitive-cost condition, the per-decoy cost regime satisfies the high-cost condition. The validation is marked as passing when the optimal decision is zero decoys under each condition and when the computed value of deception equals one under each ineffective regime.

This figure compares the ineffective-deception regimes, including the decoy-immune attacker condition and the prohibitive-cost condition, and shows the resulting optimum decoy count and VoD outcome. The figure shows that the optimal choice is zero decoys and that the value of deception equals one in each ineffective regime.

## 9. Discussion, Future Directions, and Final Thoughts

### 9.1. Discussion

The study formalizes cyber deception as a paired-game evaluation interface built around a transparent security game baseline and an augmented deceptive security game, and it treats the central scientific object as the equilibrium-measured benefit of deception relative to an equivalent non-deceptive baseline rather than as an isolated “best decoy policy” result.

**Figure 26:**
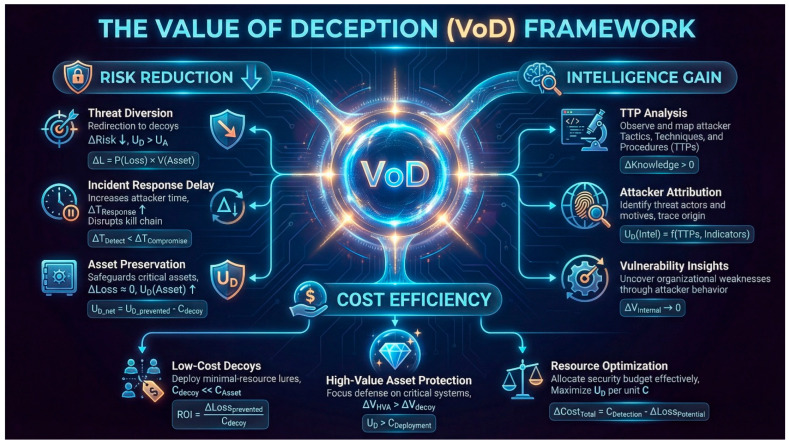
The VoD framework.

This positioning is operationalized through the value of deception, defined as a ratio of equilibrium utilities, and the price of transparency, defined as an absolute equilibrium-utility difference, with both quantities evaluated under the same strong Stackelberg equilibrium conditions, the same attacker action set, and the same linear decoy deployment cost model within the deceptive security game (Figure 26). The resulting discipline enforces comparability: a single commitment model, a single best-response and tie-breaking interpretation, and a single cost accounting convention jointly ensure that the value of deception and the price of transparency function as a coherent interface for baseline-referenced assessment rather than as disconnected metrics.

A central implication of the paired-game formulation is that deception can be analyzed through limits, thresholds, and bounds that are meaningful precisely because they are anchored to an equilibrium baseline. In this framework, curve-level summaries express how equilibrium utility changes with deployment intensity, while theorem-level results identify regimes in which deception is effective, bounded by structural limits, or collapses to the baseline.

Within this interface, the cost of deception is treated as a first-order design primitive rather than a secondary implementation detail. Theorem 8 establishes an explicit high-cost regime in which the equilibrium-optimal decision is to deploy zero decoys, and it thereby isolates a necessary feasibility condition for positive deception value under the stated accounting and equilibrium interpretation. Theorem 9 complements this regime analysis by identifying a diminishing-returns structure under which an optimal decoy count admits a marginal gain–cost threshold characterization, thereby linking curve shape to a decision rule that is interpretable under a budget constraint.

Theorem 10 extends the interface to tight performance limits by providing an upper bound on the value of deception curve under a single attacker type, a single realized attack outcome, and linear decoy deployment cost, and the associated numerical evidence clarifies when the bound is achieved and when it is conservative. Theorem 11 completes the regime characterization by identifying conditions under which deception is ineffective, including a decoy-immune attacker condition and a prohibitively high-cost condition, each of which collapses the deceptive security game back to the transparent baseline in equilibrium and yields a value of deception of one across the feasible set.

Beyond the tight-bound and characterization results, the extensions clarify operational trade-offs that recur across deception settings. In signaling-with-evidence formulations, decoy quality and detectability jointly mediate the quality–quantity trade-off under a fixed budget, so that increased realism can reduce deployment count while changing the attacker’s posterior beliefs through the detector’s operating characteristics. In dynamic rotation settings, the period choice reflects a balance between the attacker learning rate and the defender’s reconfiguration cost, so that faster attacker learning supports shorter rotation periods when rotation cost is held fixed. In bounded-rationality settings, the logit quantal response formulation provides a structured interpolation between diffuse exploration and concentrated best-response behavior, which clarifies how equilibrium-referenced measures vary as attacker rationality departs from perfect optimization.

In terms of the scope conditions that govern interpretation and transfer of the results, the payoff and detection primitives are first parameterized to support baseline-referenced comparison, and the reported curves should be interpreted as comparative objects whose operational meaning is strongest when those primitives are calibrated or bounded using controlled experiments, red-team exercises, or incident-derived measurements. Second, the use of strong Stackelberg equilibrium with strong (defender-favoring) tie-breaking provides a consistent benchmark for comparative analysis; alternative tie-breaking conventions may change point predictions in knife-edge indifference cases, but the paired-game interface remains applicable when a single convention is used consistently across the transparent and deceptive baselines.

Third, several bounds and regime results are stated under structural assumptions such as a single attacker type, a single realized attack outcome, binary detector evidence, and linear per-decoy deployment cost. These assumptions are appropriate for isolating mechanisms and deriving clean limit statements, but they also delineate the modeling scope within which each theorem should be applied. In particular, multi-stage settings with repeated attacks, richer evidence alphabets, non-linear costs, or endogenous attacker adaptation can change the mapping from deployment intensity to equilibrium response, and they motivate extensions that preserve the paired-game comparability discipline while enlarging the state and information structure.

Fourth, heterogeneous decoy allocation results are derived under a linear detection-benefit structure and, in the continuous relaxation, under assumptions that fix attacker interaction weights by decoy type. This structure yields an interpretable allocation rule under linearity, while the study’s hardness result for integer allocation formalizes why scalable relaxations and heuristic policies become necessary when integrality and heterogeneity are retained. In application, the linearity regime should therefore be treated as an analytically transparent benchmark, with richer models used when attacker interaction probabilities are expected to respond materially to portfolio composition.

Finally, the simulation and validation program is positioned as a reproducible certificate-style process designed to align computational evidence with theorem structure. For each theorem, the reported runs record the instantiated parameterization, the computed equilibrium or optimization output, and a certificate condition expressed through inequality margins, argmax identities, monotonicity statements, convergence distances, or reduction-consistency outcomes. This certificate discipline complements the analytical proofs by providing auditable numerical witnesses under the stated hypotheses, thereby strengthening the internal consistency of the equilibrium-based value of deception and price of transparency interface.

**Figure 27:**
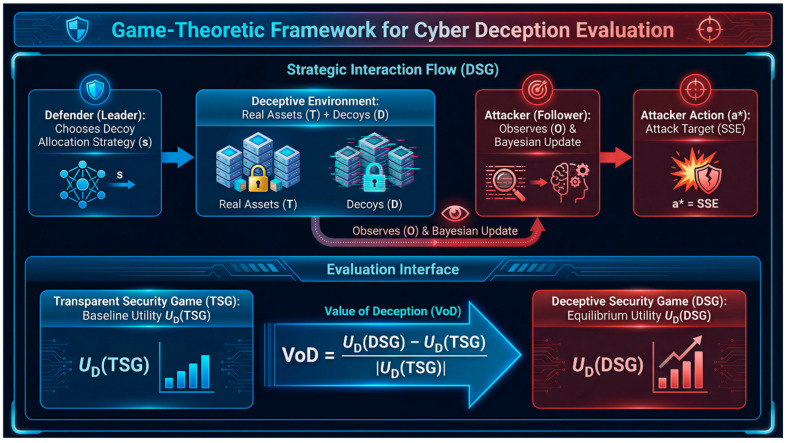
Game-theoretic framework for cyber deception evaluation.

As a whole (Figure 27), the study’s findings support the idea of evaluating cyber deception through an equilibrium-consistent, baseline-comparable interface that unifies curve-level analysis of value of deception and price of transparency with theorem-level regime characterization. The practical implications therefore emphasize disciplined comparability, cost-consistent accounting, and explicit identification of conditions under which deception is effective, bounded, or irrelevant under the stated modeling scope.

The proposed evaluation interface is designed to be implementable in operational settings where deception decisions must be updated under resource and timing constraints. The basic SDG admits enumeration-based evaluation with an explicit operation-count certificate, which supports predictable runtime scaling as the number of feasible defender strategies and attacker types grows. For larger heterogeneous catalogs (HDAP-style integer allocation), the NP-hardness result motivates practical deployment via continuous relaxations, greedy benchmarks, decomposition, or approximation-oriented routines, with solution quality judged relative to the theorem-aligned certificate outputs rather than by unverifiable optimality claims. In real deployments, latency constraints are addressed by precomputing policy tables over bounded parameter grids (e.g., cost, detector quality, attacker mixture) and performing only lightweight updates online; energy and hardware constraints can be managed by selecting solver routines consistent with the model class (LP for linear regimes; fixed-point solvers for QRE) and by using bounded-iteration stopping rules tied to residual norms that are reported as computational certificates.

#### 9.1.1. Sensitivity and Scenario Diversity Protocol

The proposed evaluation protocol treats every theoretical claim as an auditable statement whose assumptions, derived quantities, and decision rules are paired with an explicit computational certificate. Validation is therefore presented as an assumption-scoped reproducibility layer: each theorem is instantiated under its stated modeling conditions, and the corresponding target inequality, optimality condition, or limiting behavior is computed using the same primitives invoked in the theorem statement (utilities, posteriors, equilibrium best responses, or objective functions). A clear computational certificate is recorded in a form that can be re-run by an independent reader.

At the protocol level, validation is organized around four complementary elements. First, model-to-theorem fidelity is maintained by implementing each theorem’s structural ingredients without modification (e.g., discrete defender commitment sets where finiteness is assumed, Bayes posteriors where deterrence is stated in posterior form, fixed attacker interaction weights where linearity is assumed, and explicit learning-rate dynamics where rotation policies are derived). Second, certificate-based computation is applied: each claim is accompanied by a concrete artifact (objective profiles, feasibility boundaries, policy maps, equilibrium probability trajectories, linear-program extreme-point solutions, or reduction equivalence checks) documenting the evaluated statement. Third, comparative statics and limit checks are conducted when a theorem asserts monotonicity or convergence (e.g., detector-strength ordering, rationality limits, or policy shifts with learning parameters). Fourth, protocol outcomes are reported in terms of explicitly computed certificates and stated tolerances, ensuring that each theorem’s computational instantiation is documented in a consistent and reproducible manner.

Theorem 1 (existence of an optimal pure defender strategy in the finite SDG) is validated through exhaustive enumeration over the defender’s discrete strategy set (all feasible decoy counts) and direct computation of the defender objective for each candidate. The validation certificate consists of the objective profile over the finite set together with the reported maximizer, thereby documenting attainment of the optimum within the finite pure-strategy space. Theorem 2 (posterior-based deterrence feasibility and quality–quantity comparative statics under signaling-with-evidence) is validated by implementing the signaling-with-evidence model as stated, including binary evidence, a real-target false-positive rate, a quality-dependent decoy detectability function, a per-decoy cost increasing with quality, and an explicit budget constraint. The first validation step computes the Bayes posterior probability of “real” after a no-alarm observation and verifies the theorem’s deterrence condition in posterior form. The second validation step implements the comparative static by evaluating two detectability functions with pointwise ordering and confirming that, holding quality fixed, the minimal required decoy proportion is weakly larger under the more detectable decoy function; under a binding budget, the deterrence-feasible design shifts toward higher quality and lower quantity in the direction stated by the theorem.

Theorem 3 (optimal periodic rotation under attacker learning in a dynamic deception setting) is validated by instantiating a dynamic model in which the defender chooses time-indexed reconfiguration actions, the attacker’s effectiveness evolves according to an explicit learning-rate parameter, and the defender incurs a specified reconfiguration cost. A certificate is produced by solving for an optimal policy over the model’s state space using dynamic programming and recording the resulting policy and value function. The validation reports the computed optimal action structure and the corresponding policy shifts with respect to learning-rate and cost parameters, consistent with the theorem’s qualitative implications. Theorem 4 (finite-rationality bound and convergence behavior under logit QRE) is validated by computing logit quantal response choice probabilities over a sweep of the rationality parameter using a fixed-point solver and recording convergence diagnostics. The certificate compares the computed mixed strategies to the theorem’s limiting behaviors—near-uniform randomization as rationality approaches zero and concentration on best responses as rationality becomes large—within a stated numerical tolerance.

Theorem 5 (greedy/vertex allocation property in the continuous HDG relaxation under fixed interaction weights) is validated by instantiating the heterogeneous allocation model under the theorem’s linearity condition—attacker interaction probabilities with each decoy type are fixed and independent of deployed quantities—so that the defender objective is linear over a budget polytope. The certificate solves the resulting linear program and reports an optimal extreme-point allocation concentrating the budget on the decoy type identified by the theorem’s ratio rule, together with the corresponding objective value and its agreement with the linear-program optimum within tolerance. Theorem 6 (parameterized polynomial-time solvability certificate for the basic SDG under an enumeration solver) is validated by an operation-count certificate aligned with the implemented enumeration procedure: the number of feasible defender strategies is recorded, multiplied by the number of attacker types, and combined with a parameterized per-type best-response evaluation cost to yield a closed-form evaluation-count expression. The validation reports both the derived evaluation-count expression and empirical scaling behavior consistent with the predicted growth under the tested best-response cost regimes (constant-time versus linear-scan).

Theorem 7 (NP-hardness of heterogeneous integer allocation via reduction) is supported by explicitly constructing the reduction mapping used in the hardness argument and verifying decision equivalence on a suite of generated instances. The certificate maps knapsack parameters to the heterogeneous deception allocation instance parameters and evaluates feasibility for each paired instance, reporting agreement across tested cases and documenting that the instance construction scales consistently with polynomial-time mapping. Finally, cross-theorem coherence is maintained by anchoring reported outcomes to matched baseline-versus-deception constructions and by tying results to computed equilibrium quantities rather than qualitative narratives. Accordingly, the validation artifacts above are presented as a unified set of certificates showing that (i) basic SDG optimization is witnessed by explicit enumeration, (ii) leaky-deception deterrence is verified in posterior form under detector evidence, (iii) dynamic deception is evaluated through policy-level optimization under learning, (iv) bounded rationality is evaluated through a solver-verified equilibrium family with the stated limiting behaviors, (v) heterogeneous allocation yields the stated extreme-point structure under linearity assumptions, (vi) tractability claims are accompanied by an explicit operation-count certificate and scaling evidence, and (vii) intractability claims are supported by a constructed reduction and documented decision equivalence checks.

#### 9.1.2. Operational Interpretation

While the analytical and computational validations establish internal consistency within the defined game-theoretic framework, the operational interpretation of the model depends on how its primitives relate to documented cyber-attack behavior. The parameters appearing in the transparent and deception-enabled games—attack reward, deception loss, detection benefit, decoy deployment cost, detector operating characteristics, and attacker learning rate—are not abstract artifacts; each corresponds to measurable quantities that have empirical counterparts in cyber-incident reporting, red-team exercises, intrusion telemetry, and post-incident forensics.

The attacker reward parameter corresponds to the expected value of a successful compromise, which in practice can be proxied by documented breach cost estimates, asset criticality assessments, or observed attacker monetization strategies. The deception-loss parameter reflects wasted effort, exposure risk, tool burn, or operational disruption when a decoy is engaged; such quantities are observable in environments that deploy honeypots or decoy systems and record attacker dwell time, command execution attempts, and premature tool disclosure events. Detection benefit parameters can be grounded in reduced time-to-containment, improved attribution confidence, or increased probability of interrupting lateral movement, all of which are measurable through security operations center metrics and incident response records.

Similarly, detector characteristics used in the signaling-with-evidence model—false-positive rates on real assets and detectability behavior on decoys—map directly to empirical receiver operating curves of intrusion detection systems, anomaly detectors, or deception sensors deployed in operational networks. These characteristics can be estimated from labeled event data or controlled adversarial testing. In the dynamic rotation model, the attacker learning-rate parameter corresponds to empirically observable improvements in compromise efficiency over time, which can be inferred from repeated attack campaigns, time-to-compromise statistics, or adaptive behavior documented in threat intelligence reporting. Rotation costs, in turn, correspond to measured operational overhead associated with reconfiguration, redeployment, or decoy refresh procedures.

The equilibrium outcomes derived in the study, such as deterrence thresholds, diminishing returns in decoy intensity, and collapse of deception value under high transparency, are therefore interpretable as conditional predictions given empirically grounded parameter ranges. For example, if telemetry indicates that attackers rapidly distinguish low-fidelity decoys, the signaling model predicts contraction of the deterrence-feasible region unless decoy realism is increased. If incident data show slow attacker adaptation but high reconfiguration cost, the dynamic model predicts longer optimal rotation periods. These mappings illustrate that the theoretical regime characterizations are not detached abstractions; they provide structured interpretations of documented behavioral patterns under explicit cost and observability assumptions.

To make this empirical linkage explicit in deployment-oriented use, the framework supports a straightforward calibration workflow. First, a defender identifies the target class and defines payoffs consistent with operational objectives (loss avoidance, detection value, and deployment cost). Second, detector characteristics, deception interaction traces, and campaign adaptation rates are estimated from telemetry, red-team exercises, and incident documentation. Third, equilibrium quantities and reporting measures (value of deception and price of transparency) are evaluated over the calibrated ranges, yielding regime-consistent recommendations for decoy intensity, quality, and rotation cadence.

Several results in the study also adopt linear deployment costs in the number of deployed decoys, reflecting an additive cost structure in which each additional decoy contributes approximately constant marginal expense at fixed quality. This assumption is appropriate when decoys are deployed as modular artifacts with separable provisioning and monitoring costs. When deployment exhibits economies or diseconomies of scale (for example, capacity constraints, shared infrastructure, or non-linear monitoring burden), the same framework admits a direct generalization by replacing linear deployment cost with a non-linear cost function of the decoy count. Under such generalizations, equilibrium computation and posterior-based feasibility conditions remain unchanged, while optimality conditions replace constant marginal cost comparisons with marginal-cost comparisons evaluated at the relevant deployment level.

Additionally, the results that depend explicitly on linearity, most notably extreme-point allocation conclusions derived under linear objective and linear constraints—should be interpreted as structural characterizations within the linear regime. If non-linear costs or non-linear benefit accumulation are introduced, the optimization becomes a convex or mixed-integer problem depending on the chosen structure; the paired-game interface and the equilibrium-based reporting measures remain well-defined, while the allocation structure is determined by marginal-benefit-to-marginal-cost comparisons (or their discrete analogues) rather than by constant ratio rules.

The scope of the present framework is defined by a deliberate balance between analytical clarity and operational realism. The paired-game formulation isolates the strategic contribution of deception by holding the baseline targets, payoff conventions, and equilibrium rule fixed, but the resulting conclusions remain conditional on the stated modeling assumptions, including the defender-first commitment structure, the selected tie-breaking convention, and the adopted forms for costs, detectability, and attacker response. These assumptions enable clean regime characterizations and comparable equilibrium-based reporting, yet they also define the boundary within which the quantitative thresholds and optimal policies should be interpreted. The main trade-off is therefore between model richness and interpretability: richer formulations with endogenous attacker adaptation, non-linear deployment costs, repeated attacks, or more complex observation structures may capture additional operational detail, but they generally reduce tractability and weaken the availability of sharp theorem-level results. A second trade-off arises at the deployment level, where greater decoy realism, more frequent reconfiguration, and lower transparency can improve strategic value, but only at increased engineering, monitoring, and operational cost. Accordingly, the framework is best understood as a disciplined evaluative baseline that identifies how deception value changes under explicitly stated conditions, while also providing a structured foundation for future extensions that incorporate broader behavioral and deployment complexity.

### 9.2. Future Directions

Several future research directions follow directly from the study’s modeling choices and theorem assumptions, and each direction can be stated as an explicit relaxation or extension of the hypotheses used to prove and validate the results. A first direction is the systematic extension from a single attacker type and a single attack to heterogeneous attacker types and multi-attack settings while preserving the paired-game evaluation interface and the SSE interpretation. The current tight-bound and characterization results are stated under a single attacker type and single realized attack outcome, and future work can examine how VoD and PoT curves behave when attacker types are mixed, when attackers condition on partial information over time, or when multiple attacks are realized across stages with resource reallocation. This direction is naturally aligned with the study’s emphasis on curve-level analysis, because multi-stage settings would replace a single VoD(K) curve with a family of time-indexed curves or a policy-induced mapping from budget to expected utility gain under sequential play.

A second direction is the deeper integration of signaling-with-evidence structures with SSE computation in finite Stackelberg security games, so that evidence, detectability, and decoy quality enter the equilibrium computation rather than being evaluated as an auxiliary deterrence condition. The signaling-with-evidence extension already defines a Bayes posterior under a binary evidence signal and links deterrence to a type-dependent attack threshold, which creates a direct bridge between detector operating characteristics and optimal deception posture. Future work can extend this bridge by embedding evidence generation and attacker updating directly into the attacker best-response mapping under the commitment, thereby allowing VoD and PoT curves to be computed over joint choices (number of decoys, quality) that are subject to the same budget feasibility condition used in the current framework.

A third direction is the refinement of bounded rationality modeling beyond a single parametric form, while maintaining the study’s principle that all curve metrics are evaluated under the same equilibrium concept and action set. The current bounded rationality validation is framed through logit quantal response equilibrium as a fixed point across rationality levels, which is appropriate for capturing smooth departures from perfect best response. Future work can examine alternative bounded rationality mappings that preserve the defender-commitment logic but represent different attacker decision processes, and it can test which behavioral mapping best matches attacker responses in cyber deception environments where attackers face uncertainty about authenticity, evidence noise, and strategic interaction.

A fourth direction concerns the calibration and empirical grounding of payoff primitives and detection parameters. The framework is constructed to support ROI comparability across deception scenarios, and this objective becomes substantially stronger when the payoff parameters and decoy detectability functions are estimated or bounded using data from controlled experiments, red-team exercises, or operational incident records. Under such calibration, the VoD and PoT curves become not only theoretical objects but also decision-support summaries that link a defender’s budgeted deployment interface to measurable changes in expected utility under a baseline-comparable equilibrium interpretation.

A fifth direction is the expansion of the information-theoretic component into a more comprehensive “limit” analysis stated directly in terms of attacker belief uncertainty and information leakage, with explicit links to the equilibrium-based VoD/PoT interface. The study already frames a channel-style limit concept for deception stated in terms of attacker belief uncertainty and information leakage, which suggests that a joint framework can be built in which equilibrium utility gains and information-theoretic uncertainty gains are treated as coordinated outputs of the same deception design. Future work can develop conditions under which an information-theoretic improvement implies a VoD improvement under SSE, and conditions under which these notions diverge, thereby clarifying the relationship between “uncertainty induced in the attacker” and “utility improvement realized by the defender” in a baseline-comparable setting.

A sixth direction is the extension of the computational validation program to larger game instances and broader parameter regimes while preserving the certificate-style structure described in the study. The current validation process emphasizes assumption checks and solver-method alignment with theorem structure, which is a strong foundation for scaling. Future work can incorporate larger target sets, larger resource sets, richer decoy classes, and broader feasibility regimes under the same discipline: hypotheses are checked before conclusions are evaluated, and the computational object implied by each theorem is solved using a method consistent with the theorem’s formal structure.

Finally, the paired-game Stackelberg deception framework (transparent baseline versus deception-enabled counterpart, with equilibrium-referenced reporting) can be extended to Industry 5.0 smart manufacturing and cloud-based manufacturing services by treating manufacturing services, digital resources, or workflow endpoints as targets and modeling deception as protective “service-level” decoys and disguises [102]. In parallel, predictive models for financial and operational risk can inform time-varying attacker incentives and defender constraints, enabling dynamic versions of the deception game in which payoffs, costs, and transparency conditions adapt to forecasted volatility or disruption risk [103].

### 9.3. Final Thoughts and Conclusion

This study presents a unified evaluation interface for cyber deception by pairing a transparent baseline security game with a deception-enabled counterpart under a consistent equilibrium framework and cost accounting. The resulting structure supports baseline-referenced interpretation of deception value and enables consistent reporting through two complementary measures—value of deception and price of transparency—that map equilibrium utilities into operationally interpretable quantities. The analysis further extends the core formulation to settings that capture practical mechanisms affecting deception performance, including detector-mediated information leakage, periodic target rotation under attacker learning, bounded-rationality attacker response, and heterogeneous decoy portfolios with tractable structural characterization in the linear relaxation.

Across these models, the results provide a mechanism-level account of when deception increases defender value, when it exhibits diminishing returns, and when increased observability erodes effectiveness by collapsing posterior uncertainty. The computational component complements the theory by providing reproducible, certificate-oriented validations aligned with the defined decision rules and equilibrium objects, thereby strengthening internal coherence across the proposed model family. The framework thereby offers a principled foundation for subsequent empirical calibration and deployment-oriented studies that estimate detector operating characteristics, attacker behavioral parameters, and operational cost structures while retaining the same baseline-versus-deception comparability.

A natural direction for future research is to extend the present theorem-centered computational analysis with implementation-level deployment benchmarking. While the current study establishes formal complexity bounds, scalability regimes, and solver-class requirements, a next-stage investigation could complement these results with hardware-specific profiling of runtime, memory consumption, and computational overhead under representative deployment conditions. In particular, future work could benchmark the enumeration-based, dynamic-programming, fixed-point, and linear-programming components across varying problem sizes, target counts, attacker-type mixtures, and decoy portfolios, while recording wall-clock time, memory footprint, convergence behavior, and parallelization efficiency on practical computing platforms.

## Data Availability

Data are contained within the article. No dataset has been used in this work.
